# A metagenomic study of DNA viruses from samples of local varieties of common bean in Kenya

**DOI:** 10.7717/peerj.6465

**Published:** 2019-03-15

**Authors:** James M. Wainaina, Elijah Ateka, Timothy Makori, Monica A. Kehoe, Laura M. Boykin

**Affiliations:** 1 School of Molecular Sciences and Australian Research Council Centre of Excellence in Plant Energy Biology, The University of Western Australia, Crawley, WA, Australia; 2 Department of Horticulture, Jomo Kenyatta University of Agriculture and Technology, Nairobi, Kenya; 3 Diagnostic Laboratory Service, Plant Pathology, Department of Primary Industries and Regional Development, South Perth, WA, Australia

**Keywords:** Emerging viruses, Smallholder farmer, *Dicistroviridae*, Potyvirus evolutionary analysis

## Abstract

Common bean (*Phaseolus vulgaris L*.) is the primary source of protein and nutrients in the majority of households in sub-Saharan Africa. However, pests and viral diseases are key drivers in the reduction of bean production. To date, the majority of viruses reported in beans have been RNA viruses. In this study, we carried out a viral metagenomic analysis on virus symptomatic bean plants. Our virus detection pipeline identified three viral fragments of the double-stranded DNA virus *Pelargonium vein banding virus* (PVBV) (family, *Caulimoviridae*, genus *Badnavirus*). This is the first report of the *dsDNA* virus and specifically PVBV in legumes to our knowledge. In addition two previously reported *+ssRNA* viruses the bean common mosaic necrosis virus (BCMNVA) *(Potyviridae)* and aphid lethal paralysis virus (ALPV) *(Dicistroviridae)* were identified. Bayesian phylogenetic analysis of the *Badnavirus* (PVBV) using amino acid sequences of the RT/RNA-dependent DNA polymerase region showed the Kenyan sequence (SRF019_MK014483) was closely matched with two *Badnavirus* viruses: *Dracaena mottle virus (DrMV)* (YP_610965) and *Lucky bamboo bacilliform virus* (ABR01170). Phylogenetic analysis of BCMNVA was based on amino acid sequences of the Nib region. The BCMNVA phylogenetic tree resolved two clades identified as clade (I and II). Sequence from this study SRF35_MK014482, clustered within clade I with other Kenyan sequences. Conversely, Bayesian phylogenetic analysis of ALPV was based on nucleotide sequences of the hypothetical protein gene 1 and 2. Three main clades were resolved and identified as clades I–III. The Kenyan sequence from this study (SRF35_MK014481) clustered within clade II, and nested within a sub-clade; comprising of sequences from China and an earlier ALPV sequences from Kenya isolated from maize (MF458892). Our findings support the use of viral metagenomics to reveal the nascent viruses, their viral diversity and evolutionary history of these viruses. The detection of ALPV and PVBV indicate that these viruses have likely been underreported due to the unavailability of diagnostic tools.

## Introduction

Common bean (*Phaseolus vulgaris L*.) is ranked among the most important source of proteins and nutrients globally (FAO, 2018). In sub-Saharan Africa (SSA), beans constitute over 60% of all dietary proteins in each household (Katungi et al., 2009). However, significant constraints on bean production are pests and viral diseases (Ojiewo et al., 2018). The main pests present at pre-harvest include; aphids, thrips, and whiteflies (Wortmann et al., 1998). The majority of viruses reported in beans are RNA viruses of the family *Potyviridae*, in particular, bean common mosaic virus and bean common mosaic necrosis virus (BCMNVA) (Mangeni, Abang & Kelly, 2014; Worrall et al., 2015; Wainaina et al., 2018a). In Latin America, DNA viruses of the family *Geminiviridae*, which include bean golden mosaic viruses have been reported in beans (Macedo et al., 2016). The primary vector of bean golden mosaic virus is the whitefly *Bemisia tabaci*. To date, there have been no reports of any DNA viruses within common beans in SSA and in particular within the western highlands of Kenya. Therefore, given the presence of both the whitefly vector (*Bemisia tabaci*) and probable host plant (the common bean) for whitefly-transmitted viruses, we chose a high throughput sequencing approach (DNA-Seq) in an attempt to detect potential DNA viruses that might be present within the viral symptomatic beans.

The advent of high throughput sequencing (DNA-Seq and RNA-Seq) within plant virology has increased the identification of viruses through the *de novo* assembly of sequence data (Blawid, Silva & Nagata, 2017). This has subsequently led to increased detection of many understudied and novel plant viruses. There are many examples of known viruses that have been identified using these approaches (Kehoe et al., 2014; Ndunguru et al., 2015; Maina et al., 2017a; Wainaina et al., 2018a). In legumes, virus metagenomics has been used in the characterization of viruses within cowpea (Palanga et al., 2016; Jo et al., 2017; Palang et al., 2017), groundnuts (Wainaina et al., 2018b), soybean (Jo et al., 2017), common beans (Wainaina et al., 2018c) and chickpea (Leonetti et al., 2018).

Among novel viruses discovered, many have been found within crops in the tropics, including the *Badnaviruses* (family *Caulimoviridae*), which are classified as *Pararetroviruses. Badnaviruses* have been reported as an emerging threat, particularly within vegetatively propagated crops (Fargette et al., 2006; Borah et al., 2013; Iskra-Caruana et al., 2014). They affect both monocots and dicots with varying degree of symptoms from severe to mild (Bhat, Hohn & Selvarajan, 2016). Their primary mode of transmission is via insect vectors, while nematodes and fungi are marginally involved in their transmission (Hohn & Rothnie, 2013). Their primary hosts are vegetatively propagated perennial crops such as cassava (*Manihot esculenta Crantz*) and sweet potato (*Ipomoea batatas L*.) (Bousalem, Douzery & Seal, 2008; Kreuze et al., 2017) present within the agro-ecosystem in the western highlands of Kenya.

*Potyviruses* are among the most common RNA viruses found within agro-ecosystems. Species within this family are vector-transmitted, mainly by viruliferous aphids and sometimes through infected seed stock (Gibbs et al., 2008; Worrall et al., 2015). Viruses in this family have previously been reported within legumes both in the western highlands of Kenya (Mangeni, Abang & Kelly, 2014; Wainaina et al., 2018b) and within other regions globally such as the Mediterranean (Makkouk, Pappu & Kumari, 2012). Conversely another group found within legumes and aphids are the *Dicritoviridae* which are an expanding group of RNA viruses that are arthropod associated (Bonning & Miller, 2010). There genomic architecture comprises of two open reading frames (ORFs), encoded within the genome (Bonning & Miller, 2010). The *Dicritoviridae* is comprised of a single confirmed genus *Cripavirus*, which comprises of nine species. Of interest in this study is the aphid lethal paralysis virus (ALPV). ALPV is pathogenic to insects pests and has been identified in aphids (Wamonje et al., 2017) and in plants using virus metagenomic techniques (Maina et al., 2017b).

Virus metagenomics has been used for identification of viruses, and the subsequent evolutionary relationship (Pirovano et al., 2015; Roossinck, Martin & Roumagnac, 2015; Alicai et al., 2016; Maina et al., 2017a; Wainaina et al., 2018a, 2018b, 2018c). Phylogenetic relationships analysis can be established using individual genes, or the concatenated genes (Alicai et al., 2016; Wainaina et al., 2018b). However, conserved protein markers such as the RNA dependent RNA polymerase (*RdRp*) have also been used (Shi et al., 2016; Fernandez-Cassi et al., 2017). Within *+ssRNA* viruses there are several conserved viral proteins such as putative RNA helicase, chymotrypsin-like proteases, papain-like proteases, and methyltransferases have also been utilized (Koonin, Dolja & Morris, 1993; Bruenn, 2003). However, *RdRp* is highly conserved and present within a majority of the *+ssRNA* viruses and remains the reference region for most phylogenetic analysis (Bruenn, 2003; Černý et al., 2014). In addition, the coat protein gene is one of the main molecular markers both for diagnostic purpose and phylogenetic analysis (Serra-Soriano, Navarro & Pallás, 2016).

Minimal studies have been carried out to investigate the possible existence of DNA viruses circulating within common bean cultivars in the western highlands of Kenya.

We therefore carried out a high throughput (DNA-Seq) metagenomic analysis on 12 virus symptomatic common bean samples.

## Methodology

### Ethical approval and field collection

Before sample collection, ethical approval was obtained from the University of Western Australia, ethical research number (RA/4/1/7475). Fieldwork activities were coordinated through the Cassava Diagnostic project Kenyan node. Virus symptomatic common bean samples were collected as part of an extensive survey within the smallholder farms in the western highlands of Kenya as previously described (Wainaina et al., 2018c). Briefly, a total of 120 farms were sampled across the 2 years (2015/2016) during the long rain seasons (April–August). Targeted sampling technique was used to select farms with a mixed agro-ecosystem; in particular cassava and bean intercrops. The distance between any two farms was at least five km. In total, we collected *n* = 116 bean samples. Samples were stored using both silica gel and the paper press method as previously described (Abu Almakarem et al., 2012; Wainaina et al., 2018c) and transported to the BecA-ILRI Hub laboratories. DNA extraction and lyophilization were carried out, and samples were shipped to the University of Western Australia for further processing and analysis.

### DNA extraction and library preparation of the host plant

DNA extraction was carried on 12 viral leaves with chlorosis symptomatic leaves using the Quick-DNA plant/seed miniprep kit (Zymo Cat log D6020). Rolling circle amplification was then carried out on each of the DNA samples using the TempliPhi™ 100 amplification kit as described by the manufacturer (GE). Library preparation was carried out on the individual samples using the Nextera DNA library preparation kit as described by the manufacturer (Illumina, San Diego, CA, USA). Samples were dual-indexed with unique barcodes before a PCR enrichment step. For each library, quantification and determination of the correct insert size was carried out using the Agilent 2200 tape station (Agilent, Santa Clara, CA, USA) with the D1000 screen tape and ladder. Libraries with the correct insert size and concentration were sequenced with 2 × 150 bp paired-end reads on a Hiseq2500 at Macrogen, South Korea.

### Assembly of plant virome using CLC Genomic Workbench

Assembly of the sequences was based on a previously optimized method (Kehoe et al., 2014; Wainaina et al., 2018c). Briefly, de novo assembly of quality trimmed reads was carried out using the CLC Genomic Workbench (CLCGW ver. 8.5.1) with assembly parameters as previously described (Kehoe et al., 2014).

### Taxonomic profiling and virus sequence identification

**Figure 1:**
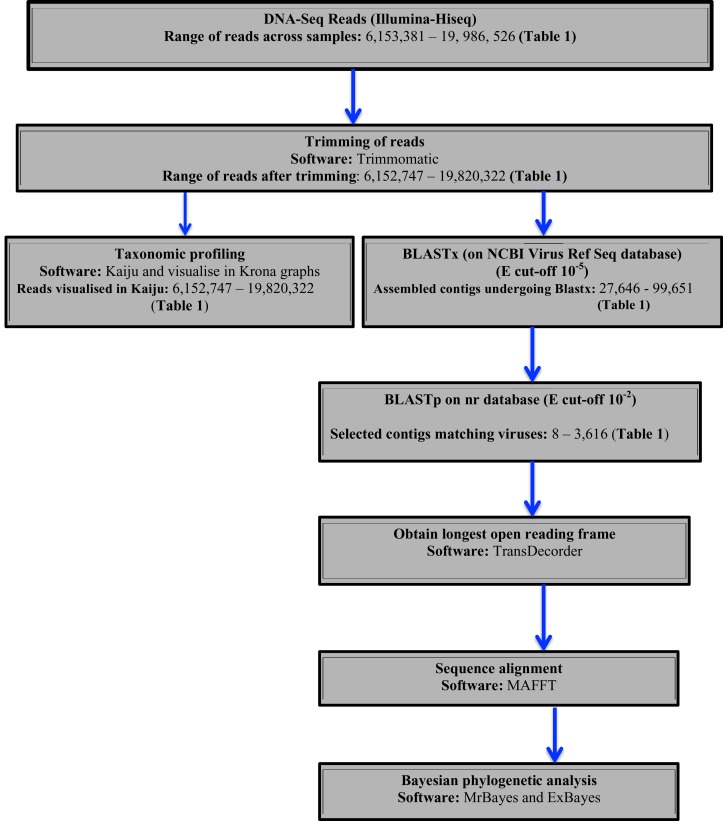
Schematic presentation and taxonomic profiling and virus identification.

Two approaches were used to identify potential viral sequences within the bean DNA-Seq reads. Taxonomic profiling was carried out on trimmed reads using Kaiju (Menzel, Ng & Krogh, 2016) using the NCBI RefSeq database containing (complete bacteria, archaea and 9,334 viral genomes) (last updated May 2017). Taxonomic results were then visualized in Krona (Ondov, Bergman & Phillippy, 2011). Secondly, assembled contigs were subjected to BLASTx searches using a preassembled viral NCBI RefSeq database (Stano, Beke & Klucar, 2016) (last updated August 2017), with an *e*-value cut of 10^−5^. Protein sequences of the top viral hits were then subjected to BLASTp search on the NCBI non-redundant protein (nr) database with an *e*-value cut-off of 10^−2^ to avoid false positives. From the confirmed viral contigs from BLASTp, the longest ORF was obtained using TransDecoder (Tang, Lomsadze & Borodovsky, 2015). The resulting amino acid sequences were then used for sequence alignment using MAFFT (Katoh & Standley, 2016) and Bayesian phylogenetic analysis using Exabayes 1.4.1 (Aberer, Kobert & Stamatakis, 2014) and MrBayes 3.2.6 (Ronquist et al., 2012). A schematic diagram of the virus identification is presented in (Fig. 1).

### Phylogenetic relationships within the viruses

Sequence alignment of amino acids of the conserved viral proteins domain was carried out using MAFFT (Katoh & Standley, 2016). Additional sequences were retrieved from GenBank, after an initial BLAST search. Within ALPV and BCMNVA all sequences from GenBank were included. In *Pelargonium vein banding virus* (PVBV) members belonging the *badnavirus* were included. For viral sequences, a consensus amino acid block without gaps was obtained using Gblock (Castresana, 2000; Dereeper et al., 2008). The amino acid sequences of the viral gene fragment were selected, as they were the gene fragments that were recovered during the viral profiling. High genetic variation within the amino acids sequences compared to the nucleotide sequences was the basis on using amino acid for the phylogenetic analysis of the viral sequences.

Bayesian phylogenetic analysis was subsequently carried out using Exabayes 1.4.1 (Aberer, Kobert & Stamatakis, 2014) and MrBayes 3.2.6 (Ronquist et al., 2012). Amino acids were assigned independent evolutionary models in Exabayes 1.4.1. ExaBayes runs were for 50 million generations using four chains. In each run, the first 25% of the sampled trees were discarded as burn-in. Convergence and mixing of the chains were evaluated using Tracer version 1.6 (Rambaut et al., 2014, http://tree.bio.ed.ac.uk/software/tracer) with effective sample size (ESS > 200) indicating convergence. The consensus tree was visualized and annotated using Fig Tree (http://tree.bio.ed.ac.uk/software/figtree). Percentage sequence similarity based on amino acid and nucleotides were estimated using the Geneious 8.1.9 (https://www.geneious.com). Sequence similarity searches as based on all BCMNVA sequences, *Badnaviruses* and all ALPV sequences in GenBank.

## Results

DNA-Seq on the 12 virus symptomatic bean samples resulted in quality-trimmed reads ranging from 6,152,747 to 19,820,322 reads (Table 1). De novo assembly using CLC genomic workbench resulted in a varying number of contigs ranging from 27,646 to 99,651 (Table 1).

**Table 1:** De novo assembly and mapping of DNA-Seq reads of common beans (*Phaseolus vulgaris L.*) reads using CLC Genomic Workbench ver. 8.5.1.

Library	GenBank Accession	No of reads before trimming	No of reads after trimming	No of contigs of assembly	N50 score	Viral contigs of interest
SRF01	SRR7264531	12,419,832	12,336,390	57,564	842	2,837
SRF02	SRR7264530	11,892,476	11,769,510	34,317	870	2,002
SRF06	SRR7264529	14,427,000	14,319,876	84,320	1,009	2,489
SRF08	SRR7264528	14,742,538	14,553,776	93,692	1,194	3300
SRF013	SRR7264535	15,150,612	15,022,436	99,651	1,174	3616
SRF019	SRR7264534	10,936,904	10,810,418	44,073	915	25
SRF020	SRR7264533	10,760,424	10,609,686	41,247	830	10
SRF021	SRR7264532	13,039,872	12,853,138	33,286	927	40
SRF022	SRR7264526	8,882,480	8,787,832	27,646	817	1,274
SRF025	SRR7264525	19,986,526	19,820,322	84,371	1185	3,268
SRF027	SRR7264524	6,153,381	6,152,747	36,009	913	8
SRF035	SRR7264527	17,832,200	13,443,658	38,342	788	1555

**Note:**

Quality assessment of the assembly was based on both the N50 scores.

### Taxonomic profiling of viruses and viral sequence confirmation

**Figure 2:**
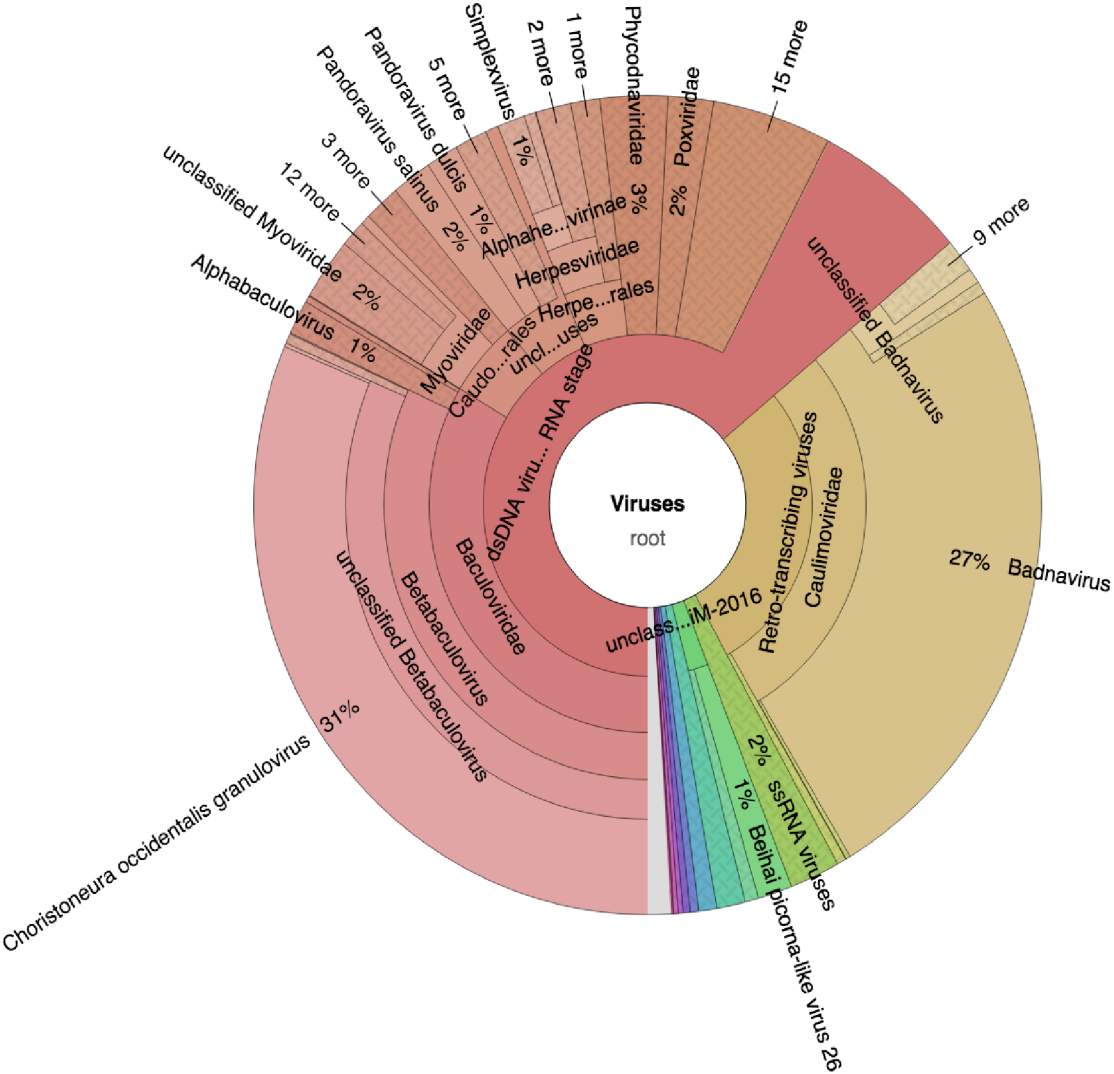
Krona graph representing DNA-Seq reads of the common bean. (*Phaseolus vulgaris L.*). Krona graph representing DNA-Seq reads of the common bean (*Phaseolus vulgaris L.*). The virus taxonomic profiles were carried out using Kaiju (Menzel, Ng & Krogh, 2016) and visualized using Krona graph (Ondov, Bergman & Phillippy, 2011). The double-stranded DNA viruses (*dsDNA*) and *ssRNA* viruses were present.

Taxonomic profiling was carried out with Kaiju (Menzel, Ng & Krogh, 2016) using the bacteria, archaea, and virus NCBI Ref-Seq. The main viruses identified were double-stranded DNA viruses such as the *Badnaviruses* that constituted at least 18% of total reads (Fig. 2; Table S1). In addition, single-stranded RNA viruses represented 2% of the total reads (Fig. 2; Table S1). Further confirmation of the viruses after assembly of the raw reads into the respective contigs (Table 1) and using a combination of sequence similarity searches using BLASTp against the non-redundant (nr) protein database confirmed three viruses (Table 2). Of the three viruses, one was a *dsDNA* virus, PVBV (family, *Caulimoviridae*, genus *Badnavirus*) and two were *+ssRNA* viruses: BCMNVA (family *Potyviridae)* and ALPV (family *Dicistroviridae)*. All raw reads from this study were deposited in GenBank with the respective accession numbers provided in Table 1.

**Table 2:** Representative sequences of the putative viral like sequences using sequence similarity searches in BLASTx and BLASTp.

Sample ID	Isolation source	Putative viral Genera	Genomic structure	Closest associated virus	% Amino and nucleotide identity	Conserved domain region detected	Fragment size	Complete genome	Sequence length	NCBI GenBank Accession	Transmission mode
SRF019 (SRR7264534)	Common bean	*Caulimoviridae* Genus *Badnavirus*	*Double stranded DNA virus*	*Pelargonium vein banding virus*	70.1	RT/RNA-dependent DNA polymerase	763	No	671	MK014483	Insect associated virus
SRF035 (SRR7264527)	Common bean	*Dicistroviridae*	*ssRNA positive-strand viruses*	*Aphid lethal paralysis virus*	95.9	Hypothesis 1 and 2 gene	815	No	815	MK014481	Aphid transmitted
		*Potyviridae*	*ssRNA positive-strand viruses*	*Bean common mosaic necrosis virus*	99.9	*RdRp*	969	No	969	MK014482	Aphid transmitted

**Note:**

Three virus sequences were identified; one double stranded DNA virus (*dsDNA*) and two +*ssRNA* viruses within symptomatic common beans.

### Bayesian phylogenetic relationship of viral sequences and nucleotide similarity

**Figure 3:**
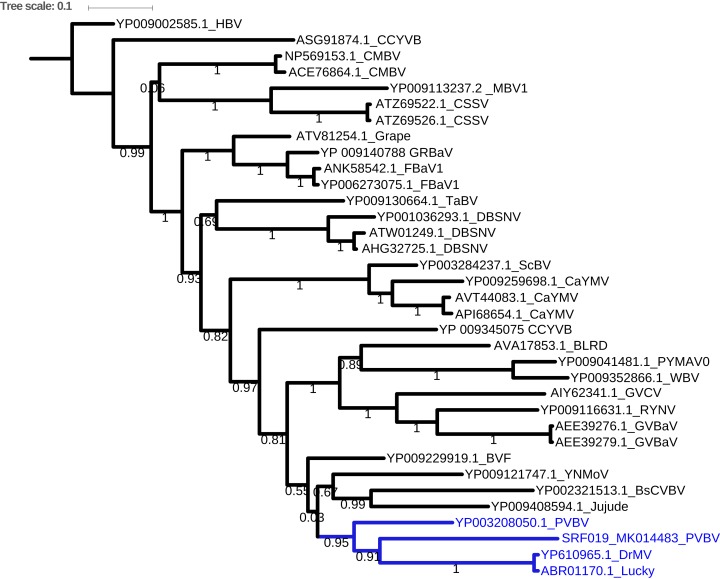
Consensus Bayesian phylogenetic tree of the *Badnavirus* using amino acid sequences of the RNA-dependent DNA polymerase region using Exabayes 1.4.1. Consensus Bayesian phylogenetic tree of the *Badnavirus* using amino acid sequences of the RNA-dependent DNA polymerase region using ExaBayes 1.4.1 (Aberer, Kobert & Stamatakis, 2014) with HBV used as the outgroup to root the tree. SRF019_SRR7264534 from this study was closest matched to the PVBV. The *Badnavirus*clade is highlighted in green. The scale bar represents amino acid subsititution per every 100 sites. The green branches represent the sequence from this study. Abbreviation: CMBV, *Citrus yellow mosaic badnavirus*; CCYVB, *Cacao yellow vein banding agent*; CSSV, *Cacao swollen shoot*; MBV-1, *Mulnerry badnavirus*; FBaV-1, *Fig badnavirus 1*; GRBaV, *Grapevine red blotch-associated virus*; TaBV, *Taro bacilliform CH virus*; Grape, *Grapevine badnavirus*; 1DBSNV, *Dioscorea bacilliform RT virus*; ScBV, *Sugarcane bacilliform*; CaYMV, *Canna yellow mottle virus*; CCYVB, *Cacao yellow vein banding*; BLRD, *Birch leaf roll-associated virus*; PYMAV0, *Pagoda yellow mosaic associated virus*; WBV1, *Wisteria badnavirus 1*; GVCV, *Grapevine vein clearing virus*; RYNV, *Rubus yellow net virus*; GVBaV, *Gooseberry vein banding associated virus*; BVF, *Blackberry* v*irus F*; YNMoV, *Yacon necrotic mottle virus*; BsCVBV, *Bougainvillea chlorotic vein banding virus*; PVBV, *Pelargonium vein banding virus*; Jujude, *Jujube mosaic-associated virus*; DrMV, *Dracaena mottle virus*; Lucky, *Lucky bamboo bacillifor mvirus*; HBV, *Hibiscus bacilliform virus*.

Bayesian phylogenetic analysis of the *Badnavirus* using amino acid sequences of the RNA-dependent DNA polymerase gene region showed the Kenyan sequence (SRF019_MK014481) was clustered with two viruses: *Dracaena mottle virus (DrMV)* (YP_610965) and *Lucky bamboo bacilliform virus* (ABR01170) (Fig. 3).

**Figure 4:**
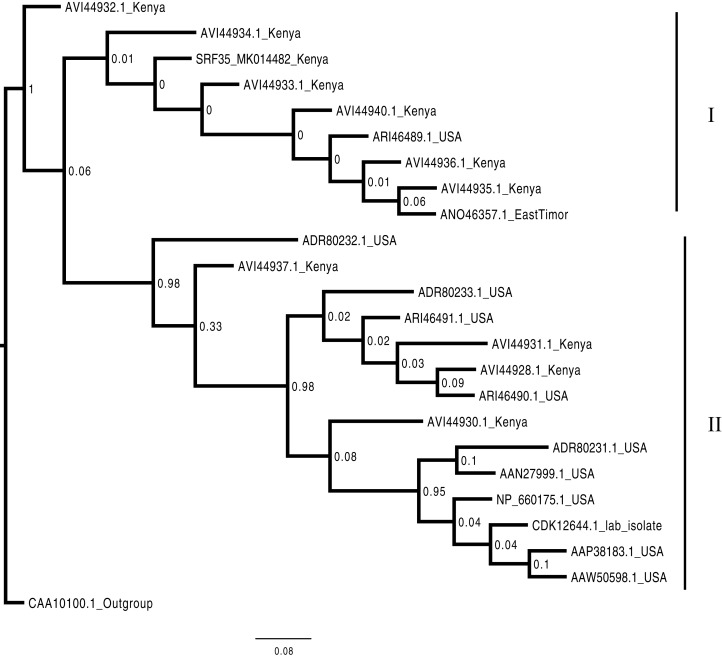
Consensus Bayesian phylogenetic tree of the Bean common mosaic necrosis virus amino acid (BCMNVA) sequence of the Nib, using Exabayes 1.4.1. Consensus Bayesian phylogenetic tree of the Bean common mosaic necrosis virus amino acid (BCMNVA) sequence of the Nib, using ExaBayes 1.4.1 (Aberer, Kobert & Stamatakis, 2014) with the Bean common mosaic virus (BCMV) used as the outgroup. Two clades were resolved within the phylogenetic tree identified as clades I and II. Sequences from this study, SRF35_SRR7264527 clustered within the clade I with other BCMNVA sequences previously reported from Kenya. The BCMNVA clade is highlighted in green. The scale bar represents amino acid substitution per every 100 sites. Abbreviation: BCMNVA, Bean common mosaic necrosis virus; BCMV, Bean common mosaic virus.

**Figure 5:**
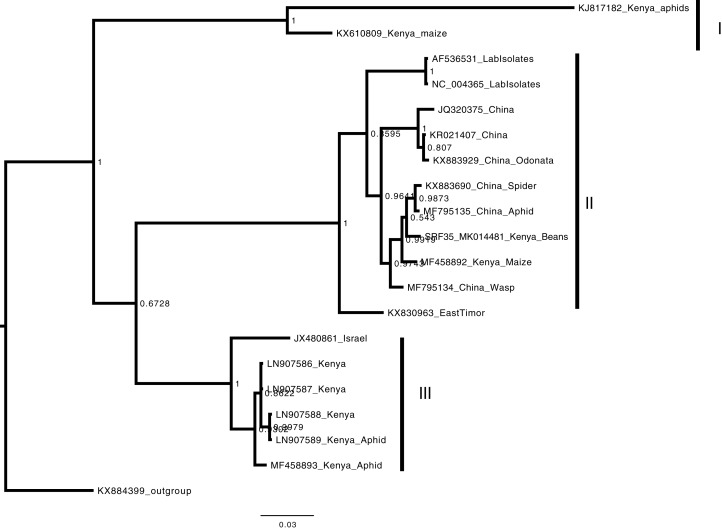
Consensus Bayesian phylogenetic tree of *Aphid lethal paralysis virus* (ALPV) using nucleotide sequences of the hypothetical protein gene 1 and 2 using MrBayes 3.2.6. Consensus Bayesian phylogenetic tree of *Aphid lethal paralysis virus* (ALPV) using nucleotide sequences of the hypothetical protein gene 1 and 2 using MrBayes 3.2.6 (Aberer, Kobert & Stamatakis, 2014). *Hubei picorna-like virus* (KX884399) was used as the out-group to root the phylogenetic tree. Three well-supported clades were resolved and identified as clades I–III. The Kenyan sequence SRF35_SRR7264527 was clustered in clade II within a nest sub clade comprising of sequences from previously identified from China to Kenya. The scale bar represents the amino acid substitution per every 100 sites.

Phylogenetic analysis of the BCMNVA based on the amino acid sequences of the Nib, gene, resolved two main clades (Fig. 4). Sequence from this study (SRF35_MK014482) clustered within clade I with previously reported BCMNVA sequences (Fig. 4). Bayesian phylogenetic analysis of ALPV was based on nucleotide sequences of the hypothetical protein gene 1 and 2. Three main clades were resolved and identified as clades I–III (Fig. 5). The Kenyan sequence from this study (SRF35_MK014481) clustered within clade II and within a nested sub-clade. This nested sub-clade comprised of sequences from China isolated from various insects (wasp, aphids, and spiders) in addition to another ALPV sequence from Kenya isolated from maize (MF458892) (Fig. 5). Percentage sequence similarity of the hypothetical protein 1 and 2 matched the Kenyan sequence (SRF019_MK014483) to PVBV GenBank sequence (YP_003208050.1) with 70.5% sequence similarity (Table S2). In ALPV nucleotide sequences of the hypothetical protein 1 and 2 from this study (SRF35_MK014481) were closely matched with sequences with 99% from China and isolated from aphids (MF795135.1) from GenBank (Table S3). For BCMNVA the percentage sequence similarity was based on amino acid sequences of the Nib region, which closely matched the Kenyan sequence (SRF35_MK014482) to a GenBank sequence with 100% identity matched to a previously isolated Kenyan sequence (AV144940_Kenya) (Table S4). Sequence alignments within the three gene fragments are presented in Figs. S1–S3.

## Discussion

Heterogeneous agro-ecosystems of the western highlands of Kenya have been reported to have a wide range of viruses in circulation (Mangeni, Abang & Kelly, 2014; Wainaina et al., 2018a; Wainaina et al., 2018b). The viruses reported so far within the beans in this region have mainly been RNA viruses (Mangeni, Abang & Kelly, 2014; Wainaina et al., 2018a, 2018b). To date, there are have been no studies on potential DNA viruses within common beans in the western highlands of Kenya. We report the first evidence of PVBV, a DNA viral sequence in beans, and an additional two RNA viruses BCMNVA and ALPV previously reported from this region (Wamonje et al., 2017; Wainaina et al., 2018c). Moreover, we establish the evolutionary relationship of these three viruses and compare them with previously reported sequences from GenBank.

Assessment of the DNA viruses within bean cultivars was carried out using a viral metagenomic approach (DNA-Seq). Taxonomic profiling identified both *dsDNA* and *ssRNA* viruses (Fig. 2). Further confirmation using sequence similarity searches nr BLASTp identified three main viruses (Table 2). Among these viruses are the *dsDNA* viruses from the genus *Badnavirus*, the PVBV (Table 2). There have been reports of *Badnaviruses* within legumes such as the peanut chlorotic stunt virus and the soybean mild mottle virus (De Kochko et al., 1998). *Badnaviruses* are transmitted in a semi-persistent mode by the insect vectors. Aphids and whiteflies have been implicated in the transmission of different viral species within the genus *Badnavirus.* In addition, the use of cuttings could also be a driver of the continued presence of *Badnaviruses* such as the Sweet potato badnavirus within the agro-ecosystems (Kreuze et al., 2017). It is therefore likely that the infection of *Badnavirus* within beans could be due to feeding by viruliferous aphids and whiteflies. Further evidence of this was the presence of the *Potyvirus* BCMNVA (Table 2; Fig. 3). This virus is mainly transmitted by aphids and by seed-borne transmission (Worrall et al., 2015). BCMNVA has previously been reported as one of the most prevalent viruses within common beans in the western highlands of Kenya (Mangeni, Abang & Kelly, 2014; Wainaina et al., 2018a). Both the recycling of infected seeds from one season to the next and the presence of viruliferous aphid vectors are thought to be the primary mode of transmission of BCMNVA in the western highlands of Kenya (Mangeni, Abang & Kelly, 2014; Wainaina et al., 2018a). This, therefore, supports our hypothesis that both BCMNVA and PVBV within the beans could be obtained from aphids that were foraging across the different plants as they search for their preferred host plant. This is especially so within smallholder farms where there are multiple host plants within the agro-ecosystem. We postulate as the aphids move from one crop to another, they inoculate these viral particles within the alternative host plants which act as a virus sink within the cropping system.

A limitation of our study was that though RNase treatment was conducted, we think the treatment was inadequate. Hence we identified both DNA and RNA viruses from our samples that we subsequently analyzed. These findings further demonstrate the abundance of RNA transmitted viruses in legumes (Wainaina et al., 2018a), and the power of metagenomic studies for virus discovery. Moreover, it could also be that since we did not obtain complete genomes of either of these viruses and obtained only viral fragments it is probable that the viral fragment in particular from the RNA the virus could have integrated within the host plant. Integration of viruses within host genome has been previously reported, and are considered as viral fossils (Geering et al., 2014). However, we could not ascertain this due to a lack of biological samples to conduct more extensive sequencing with a high sequencing coverage depth to resolve this problem.

The presence of the arthropod (i.e., aphid) infecting virus the (ALPV) (Bonning & Miller, 2010; Liu et al., 2014) further supports the hypothesis of beans as viral sinks or sources (Fereres, 2000; Uzokwe et al., 2016). The ALPV sequence identified in this study (SRF35_MK014481) was closely related to previous Kenyan sequence isolated from maize (MF458892) and sequences from China (Fig. 4). Our findings are comparable with previous studies such as those conducted by Granberg et al. (2013), Wamonje et al. (2017), and Maina et al. (2017b), where ALPV has been isolated in different host plants (maize, beans) and insects (aphids and honey bee). ALPV is transmitted between insects through both horizontal and vertical modes of transmission (Bonning & Miller, 2010). We hypothesize that ALPV and the host plants (beans) exist in a mutualistic relationship. Based on our hypothesis, of beans acting as viral sources and/or sinks, beans ensure the survival of ALPV by acting as pseudo hosts, as ALPV moves between insect vectors. In exchange, ALPV infects aphids infesting beans making them susceptible to ladybirds and the parasitoid wasp *Aphidius ervi* Ban et al. (2008). We posit that this may reduce the aphid load on the bean plants (Wamonje et al., 2017). Moreover, Verbeek et al. (2002) demonstrated that ALPV could also readily infect whiteflies. It is thus likely that infection of whiteflies by ALPV might make them more susceptible to predators of the whitefly *Bemisia tabaci* and *Trialeurodes vaporariorum* that have previously been reported (Macfadyen et al., 2018). From our field observations, beans were less infested with whiteflies compared to cassava and sweet potato within the same agro-ecosystem. The potential role of viruses, in particular, those of the family *Dicistroviridae* as biocontrol agents of insect vectors in plants, warrants further investigation. Biocontrol strategies of insects within smallholder farmers could be readily applicable due to the relatively low cost to smallholder farmers.

## Conclusion

In this study, we used a viral metagenomic (DNA-Seq) approach to identify viruses within symptomatic viral beans. We identified viruses of the family *Caulimoviridae*, genus *Badnavirus*, the PVBV. Moreover we detected *+ssRNA* viruses of family *Potyviruses* BCMNVA. In addition, an arthropod infecting virus from the family *Dicistroviridae*, ALPV was also identified. The beans were infected with both plant and arthropod infecting viruses. This could further suggest that beans are potential viral sources or sinks within heterogeneous agro-ecosystems. Future studies could complement our findings assessing the potential role of arthropod infecting viruses as potential biocontrol agents within infestation in legumes and other crops found within the agro-ecosystem.

## Supplemental Information

10.7717/peerj.6465/supp-1 Supplemental Information 1 Table S1. Summary of the taxonomic profile of the DNA-Seq reads of the 12 bean samples using Kaiju (Menzel, Ng & Krogh, 2016) (raw data).

10.7717/peerj.6465/supp-2 Supplemental Information 2 Table S2. Pairwise comparison of the amino acids sequence alignment of the Pelargonium vein-banding virus (PVBV).

10.7717/peerj.6465/supp-3 Supplemental Information 3 Table S3. Pairwise comparison of the nucleotide sequence alignment of the Aphid lethal paralysis virus (ALPV).

10.7717/peerj.6465/supp-4 Supplemental Information 4 Table S4. Pairwise comparison of the amino acids sequence alignment of the Bean common mosaic necrosis virus.

10.7717/peerj.6465/supp-5 Supplemental Information 5 Fig. S1. Alignment of PVBV. Sequence alignment of amino acid sequences of the RT/RNA-dependent DNA polymerase region of the Badnavirus using MAFFT (Katoh & Standley, 2016).

10.7717/peerj.6465/supp-6 Supplemental Information 6 Fig. S2. Alignment of BCMNV. Sequence alignment of the amino acid sequences of the RdRp region of Bean common mosaic necrosis virus using MAFFT (Katoh & Standley, 2016).

10.7717/peerj.6465/supp-7 Supplemental Information 7 Fig. S3. Sequence alignment nucleotide sequences of the hypothetical protein 1 and 2 region of the Aphid lethal paralysis virus using MAFFT (Katoh & Standley, 2016).

## Additional Information and Declarations

### Field Study Permissions

The following information was supplied relating to field study approvals (i.e., approving body and any reference numbers): Before sample collection, ethical approval was obtained from the University of Western Australia (UWA), ethical research number (RA/4/1/7475).

### Data Availability

The following information was supplied regarding data availability: Short reads were deposited in NCBI short read archive under accession numbers: SRR7264527—SRR7264531.
