# Supplementary material for: A metagenomic study of DNA viruses from samples of local varieties of common bean in Kenya
Source: PeerJ. 2019 Mar 15;7:e6465. doi: 10.7717/peerj.6465 (PMC6422016; doi:10.7717/peerj.6465)
Supplement: Supplemental Information 5 — Sequence alignment of amino acid sequences of the RT/RNA-dependent DNA polymerase region of the Badnavirus using MAFFT (Katoh & Standley, 2016). [file peerj-07-6465-s005.pdf]

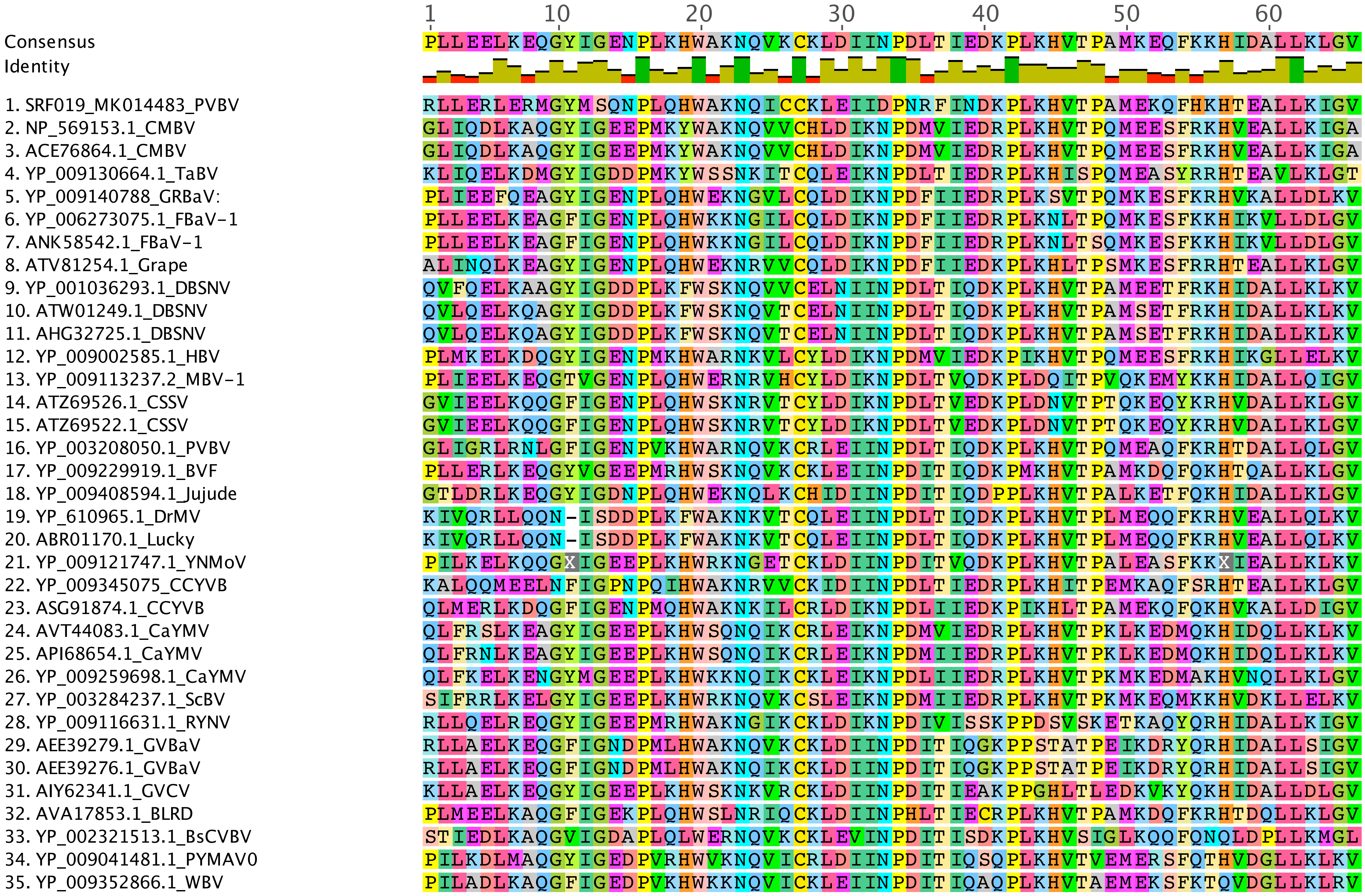

1 10 20 30 40 50 60
Consensus PLLEELKEQGYIGENPLKHWAKNQVKCKLDIINPDLTIEDKPLKHVTPAMKEQFKKHIDALLKLGV
Identity
1. SRF019_MK014483_PVBV RLLERLERMGYMSQNPLQHWAKNQICCKLEIIDPNRFINDKPLKHVTPAMEKQFHKHTEALLKIGV
2. NP_569153.1_CMBV GLIQDLKAQGYIGEEPMKYWAKNQVVCHLDIKNPDMVIEDRPLKHVTPQMEESFRKHVEALLKIGA
3. ACE76864.1_CMBV GLIQDLKAQGYIGEEPMKYWAKNQVVCHLDIKNPDMVIEDRPLKHVTPQMEESFRKHVEALLKIGA
4. YP_009130664.1_TaBV KLIQELKDMGYIGDDPMKYWSSNKITCQLEIKNPDLTIEDRPLKHISPQMEASYRRHTEAVLKLGT
5. YP_009140788_GRBaV: PLIEEFQEAGYIGENPLQHWEKNGVLCQLDIKNPDFIIEDRPLKSVTPQMKESFKRHVKALLDLKV
6. YP_006273075.1_FBaV-1 PLLEELKEAGFIGENPLQHWKKNGILCQLDIKNPDFIIEDRPLKNLTPQMKESFKKHIKVLLDLGV
7. ANK58542.1_FBaV-1 PLLEELKEAGFIGENPLQHWKKNGILCQLDIKNPDFIIEDRPLKNLTSQMKESFKKHIKVLLDLGV
8. ATV81254.1_Grape ALINQLKEAGYIGENPLQHWEKNRVVCQLDIKNPDFIIEDKPLKHLTPSMKESFRRHTEALLKLGV
9. YP_001036293.1_DBSNV QVFQELKAAGYIGDDPLKFWSKNQVVCELNIINPDLTIQDKPLKHVTPAMEETFRKHIDALLKLKV
10. ATW01249.1_DBSNV QVLQELKQAGYIGDDPLKFWSKNQVTCELNIINPDLTIQDKPLKHVTPAMSETFRKHIDALLKLKV
11. AHG32725.1_DBSNV QVLQELKQAGYIGDDPLKFWSKNQVTCELNIINPDLTIQDKPLKHVTPAMSETFRKHIDALLKLKV
12. YP_009002585.1_HBV PLMKELKDQGYIGENPMKHWARNKVLCYLDIKNPDMVIEDKPIKHVTPQMEESFRKHIKGLLELKV
13. YP_009113237.2_MBV-1 PLIEELKEQGTVGENPLQHWERNRVHCYLDIKNPDLTVQDKPLDQITPVQKEMYKKHIDALLQIGV
14. ATZ69526.1_CSSV GVIEELKQQGFIGENPLQHWSKNRVTCYLDIKNPDLTVEDKPLDNVTPTQKEQYKRHVDALLKLGV
15. ATZ69522.1_CSSV GVIEELKQQGFIGENPLQHWSKNRVTCYLDIKNPDLTVEDKPLDNVTPTQKEQYKRHVDALLKLGV
16. YP_003208050.1_PVBV GLIGRLRNLGFIGENPVKHWARNQVKCRLEIINPDLTIQDKPLKHVTPQMEAQFKRHTDALLQLGV
17. YP_009229919.1_BVF PLLERLKEQGYVGEEPMRHWSKNQVKCKLEIINPDITIQDKPMKHVTPAMKDQFQKHTQALLKLGV
18. YP_009408594.1_Jujude GTLDRLKEQGYIGDNPLQHWEKNQLKCHIDIINPDITIQDPPLKHVTPALKETFQKHIDALLKLGV
19. YP_610965.1_DrMV KIVQRLLQQN-ISDDPLKFWAKNKVTCQLEIINPDLTIQDKPLKHVTPLMEQQFKRHVEALLQLKV
20. ABR01170.1_Lucky KIVQRLLQQN-ISDDPLKFWAKNKVTCQLEIINPDLTIQDKPLKHVTPLMEQQFKRHVEALLQLKV
21. YP_009121747.1_YNMoV PILKELKQQGXIGEEPLKHWRKNGETCKLDIINPDITVQDKPLKHVTPALEASFKKXIEALLKLKV
22. YP_009345075_CCYVB KALQQMEELNFIGPNPQIHWAKNRVVCKIDIINPDLTIEDRPLKHITPEMKAQFSRHTEALLKLGV
23. ASG91874.1_CCYVB QLMERLKDQGFIGENPMQHWAKNKILCRLDIKNPDLIIEDKPIKHLTPAMEKQFQKHVKALLDIGV
24. AVT44083.1_CaYMV QLFRSLKEAGYIGEEPLKHWSQNQIKCRLEIKNPDMVIEDRPLKHVTPKLKEDMQKHIDQLLKLKV
25. API68654.1_CaYMV QLFRNLKEAGYIGEEPLKHWSQNQIKCRLEIKNPDMIIEDRPLKHVTPKLKEDMQKHIDQLLKLKV
26. YP_009259698.1_CaYMV QLFKELKENGYMGEEPLKHWKKNQIKCKLEIKNPDLIIEDRPLKHVTPKMKEDMAKHVNQLLKLGV
27. YP_003284237.1_ScBV SIFRRLKELGYIGEEPLKHWRKNQVKCSLEIKNPDMIIEDRPLKHVTPKMKEQMKKHVDKLLELKV
28. YP_009116631.1_RYNV RLLQELREQGYIGEEPMRHWAKNGIKCKLDIKNPDIVISSKPPDSVSKETKAQYQRHIDALLKIGV
29. AEE39279.1_GVBaV RLLAELKEQGFIGNDPMLHWAKNQVKCKLDIINPDITIQGKPPSTATPEIKDRYQRHIDALLSIGV
30. AEE39276.1_GVBaV RLLAELKEQGFIGNDPMLHWAKNQIKCKLDIINPDITIQGKPPSTATPEIKDRYQRHIDALLSIGV
31. AIY62341.1_GVCV KLLAELKEQGYIGEEPLKHWSKNKVRCKLDIINPDITIEAKPPGHLTLEDKVKYQKHIDALLDLGV
32. AVA17853.1_BLRD PLMEELKAQGFIGEKPLQHWSLNRIQCKLDIINPHLTIECRPLKHVTPAMKDQFKRHTDQLLKLGV
33. YP_002321513.1_BsCVBV STIEDLKAQGVIGDAPLQLWERNQVKCKLEVINPDITISDKPLKHVSIGLKQQFQNQLDPLLKMGL
34. YP_009041481.1_PYMAV0 PILKDLMAQGYIGEDPVRHWVKNQVICRLDIINPDITIQSQPLKHVTVEMERSFQTHVDGLLKLKV
35. YP_009352866.1_WBV PILADLKAQGFIGEDPVKHWKKNQVICKLEIINPDITIQAQPLKHVTAEMEKSFKTQVDGLLKLRV

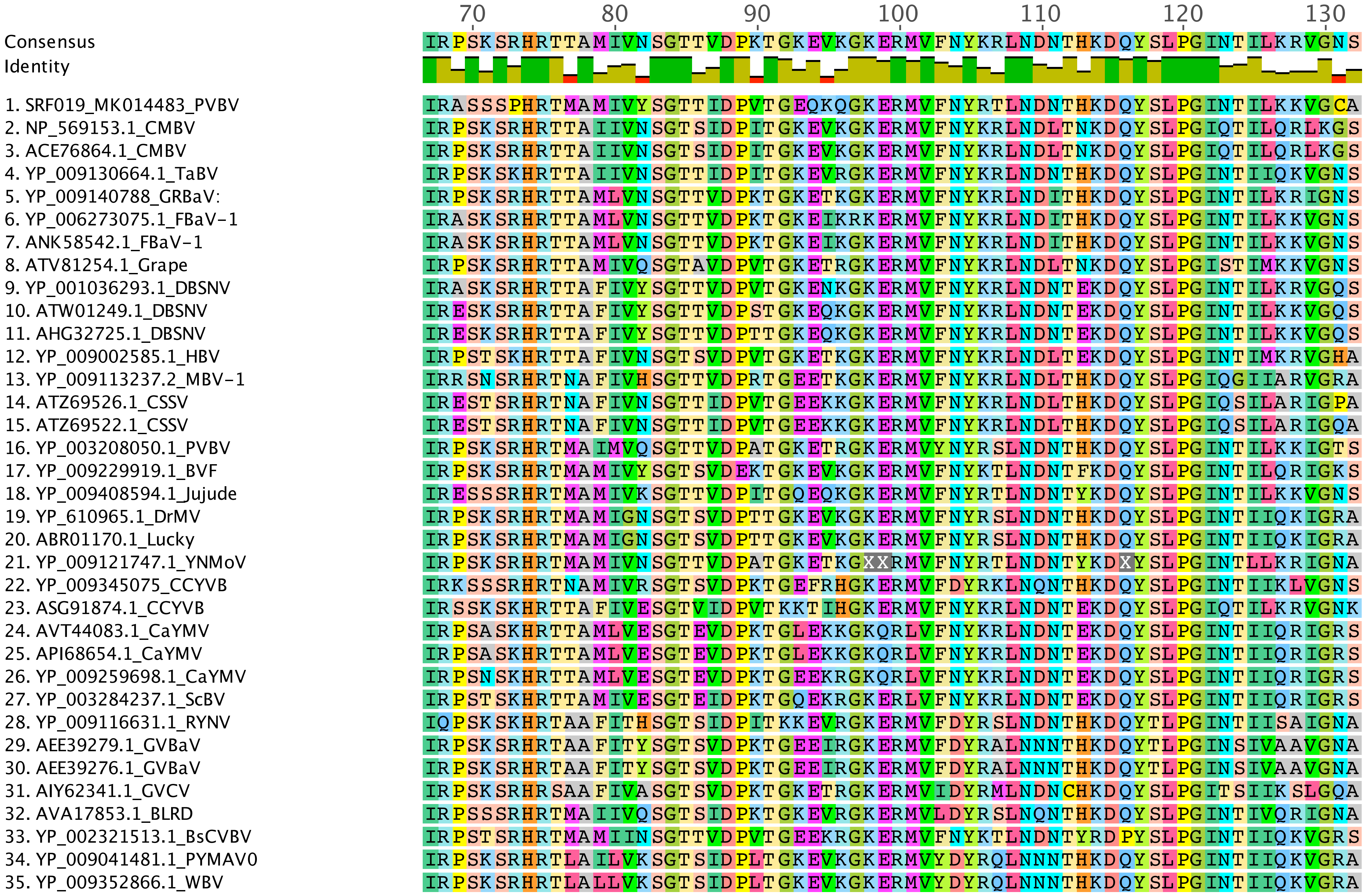
70 80 90 100 110 120 130
Consensus IRPSKSRHRTTAMIVNSGTTVDPKTGKEVKGKERMVFNYKRLNDNTHKDQYSLPGINTILKRVGNS
Identity
1. SRF019_MK014483_PVBV IRASSSPHRTMAMIVYSGTTIDPVTGEQKQGKERMVFNYRTLNDNTHKDQYSLPGINTILKKVGCA
2. NP_569153.1_CMBV IRPSKSRHRTTAIIVNSGTSIDPITGKEVKGKERMVFNYKRLNDLTNKDQYSLPGIQTILQRLKGS
3. ACE76864.1_CMBV IRPSKSRHRTTAIIVNSGTSIDPITGKEVKGKERMVFNYKRLNDLTNKDQYSLPGIQTILQRLKGS
4. YP_009130664.1_TaBV IRPSKSKHRTTAIIVNSGTTIDPITGKEVRGKERMVFNYKRLNDNTHKDQYSLPGINTIIQKVGNS
5. YP_009140788_GRBaV: IRPSKSRHRTTAMLVNSGTTVDPKTGKETKGKERMVFNYKRLNDITHKDQYSLPGINTILKRIGNS
6. YP_006273075.1_FBaV-1 IRASKSRHRTTAMLVNSGTTVDPKTGKEIKRKERMVFNYKRLNDITHKDQYSLPGINTILKKVGNS
7. ANK58542.1_FBaV-1 IRASKSRHRTTAMLVNSGTTVDPKTGKEIKGKERMVFNYKRLNDITHKDQYSLPGINTILKKVGNS
8. ATV81254.1_Grape IRPSKSRHRTTAMIVQSGTAVDPVTGKETRGKERMVFNYKRLNDLTNKDQYSLPGISTIMKKVGNS
9. YP_001036293.1_DBSNV IRASKSRHRTTAFIVYSGTTVDPVTGKENKGKERMVFNYKRLNDNTEKDQYSLPGINTILKRVGQS
10. ATW01249.1_DBSNV IRESKSRHRTTAFIVYSGTTVDPSTGKEQKGKERMVFNYKRLNDNTEKDQYSLPGINTILKKVGQS
11. AHG32725.1_DBSNV IRESKSRHRTTAFIVYSGTTVDPTTGKEQKGKERMVFNYKRLNDNTEKDQYSLPGINTILKKVGQS
12. YP_009002585.1_HBV IRPSTSKHRTTAFIVNSGTSVDPVTGKETKGKERMVFNYKRLNDLTEKDQYSLPGINTIMKRVGHA
13. YP_009113237.2_MBV-1 IRRSNSRHRTNAFIVHSGTTVDPRTGEETKGKERMVFNYKRLNDLTHKDQYSLPGIQGIIARVGRA
14. ATZ69526.1_CSSV IRESTSRHRTNAFIVNSGTTIDPVTGEEKKGKERMVFNYKRLNDLTHKDQYSLPGIQSILARIGPA
15. ATZ69522.1_CSSV IRESTSRHRTNAFIVNSGTTIDPVTGEEKKGKERMVFNYKRLNDLTHKDQYSLPGIQSILARIGQA
16. YP_003208050.1_PVBV IRPSKSRHRTMAIMVQSGTTVDPATGKETRGKERMVYNYRSLNDNTHKDQYSLPGINTILKKIGTS
17. YP_009229919.1_BVF IRPSKSRHRTMAMIVYSGTSVDEKTGKEVKGKERMVFNYKTLNDNTFKDQYSLPGINTILQRIGKS
18. YP_009408594.1_Jujude IRESSSRHRTMAMIVKSGTTVDPITGQEQKGKERMVFNYRTLNDNTYKDQYSLPGINTILKKVGNS
19. YP_610965.1_DrMV IRPSKSRHRTMAMIGNSGTSVDPTTGKEVKGKERMVFNYRSLNDNTHKDQYSLPGINTIIQKIGRA
20. ABR01170.1_Lucky IRPSKSRHRTMAMIGNSGTSVDPTTGKEVKGKERMVFNYRSLNDNTHKDQYSLPGINTIIQKIGRA
21. YP_009121747.1_YNMoV IRPSKSRHRTMAMIVNSGTTVDPATGKETKGXXRMVFNYRTLNDNTYKDXYSLPGINTLLKRIGNA
22. YP_009345075_CCYVB IRKSSSRHRTNAMIVRSGTSVDPKTGEFRHGKERMVFDYRKLNQNTHKDQYSLPGINTIIKLVGNS
23. ASG91874.1_CCYVB IRSSKSKHRTTAFIVESGTVIDPVTKKTIHGKERMVFNYKRLNDNTEKDQYSLPGIQTILKRVGNK
24. AVT44083.1_CaYMV IRPSASKHRTTAMLVESGTEVDPKTGLEKKGKQRLVFNYKRLNDNTEKDQYSLPGINTIIQRIGRS
25. API68654.1_CaYMV IRPSASKHRTTAMLVESGTEVDPKTGLEKKGKQRLVFNYKRLNDNTEKDQYSLPGINTIIQRIGRS
26. YP_009259698.1_CaYMV IRPSNSKHRTTAMLVESGTEVDPKTGEEKRGKQRLVFNYKRLNDNTEKDQYSLPGINTIIQRIGRS
27. YP_003284237.1_ScBV IRPSTSKHRTTAMIVESGTEIDPKTGQEKRGKERLVFNYKRLNDNTEKDQYSLPGINTIIQRIGRS
28. YP_009116631.1_RYNV IQPSKSKHRTAAFITHSGTSIDPITKKEVRGKERMVFDYRSLNDNTHKDQYTLPGINTIISAIGNA
29. AEE39279.1_GVBaV IRPSKSRHRTAAFITYSGTSVDPKTGEEIRGKERMVFDYRALNNNTHKDQYTLPGINSIVAAVGNA
30. AEE39276.1_GVBaV IRPSKSRHRTAAFITYSGTSVDPKTGEEIRGKERMVFDYRALNNNTHKDQYTLPGINSIVAAVGNA
31. AIY62341.1_GVCV IRPSKSRHRSAAFIVASGTSVDPKTGKETRGKERMVIDYRMLNDNCHKDQYSLPGITSIIKSLGQA
32. AVA17853.1_BLRD IRPSSSRHRTMAIIVQSGTSIDPKTGKEVRGKERMVLDYRSLNQNTHKDQYSLPGINTIVQRIGNA
33. YP_002321513.1_BsCVBV IRPSTSRHRTMAMIINSGTTVDPVTGEEKRGKERMVFNYKTLNDNTYRDPYSLPGINTIIQKVGRS
34. YP_009041481.1_PYMAV0 IRPSKSRHRTLAILVKSGTSIDPLTGKEVKGKERMVYDYRQLNNNTHKDQYSLPGINTIIQKVGRA
35. YP_009352866.1_WBV IRPSKSRHRTLALLVKSGTSIDPLTGKEVKGKERMVYDYRQLNNNTHKDQYSLPGINTIIQKVGRA

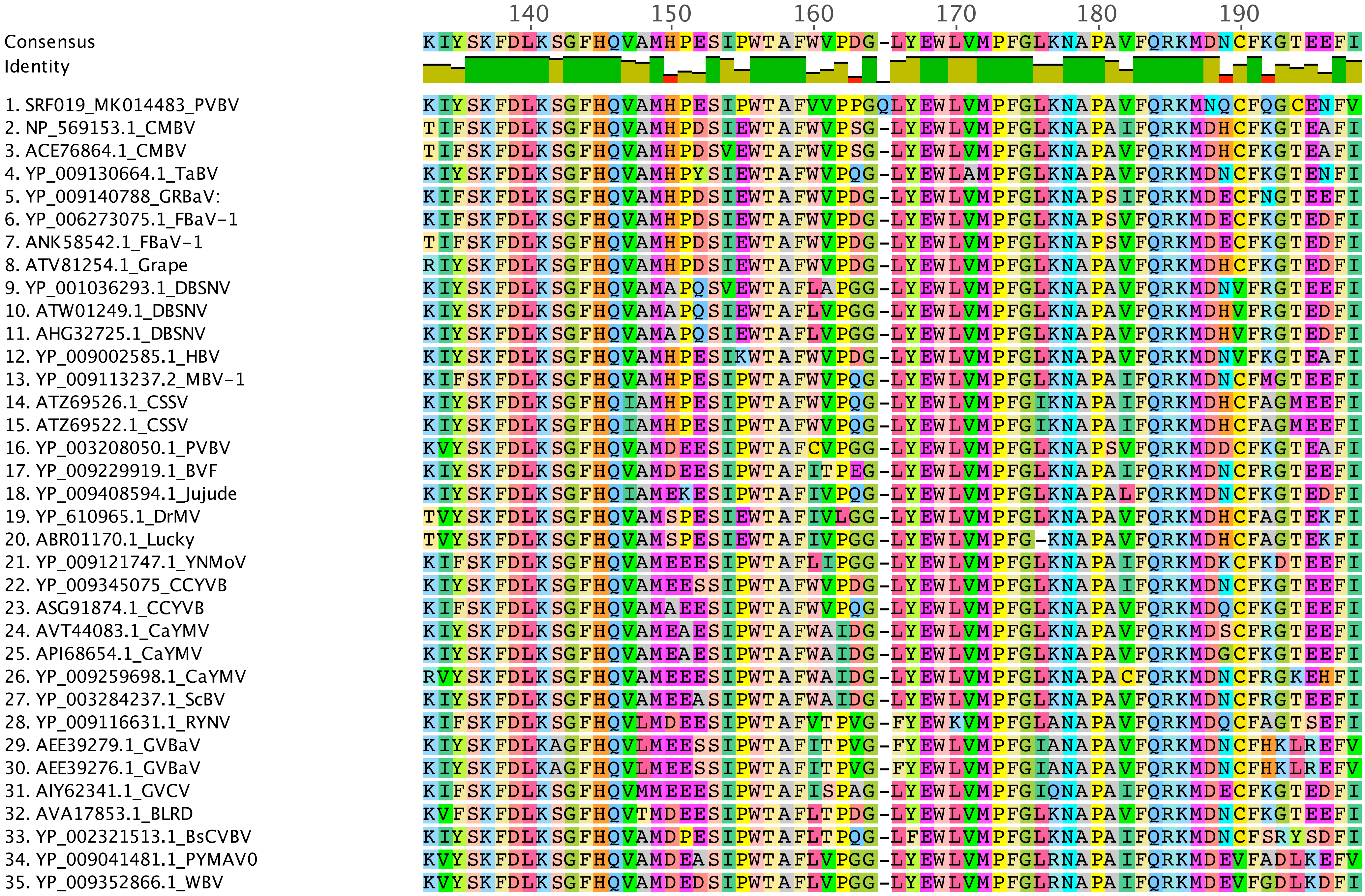

140
150
160
170
180
190
Consensus KIYSKFDLKSGFHQVAMHPESIPWTAFWVPDG-LYEWLVMPFGLKNAPAVFQRKMDNCFKGTEEFI
Identity
1. SRF019_MK014483_PVBV KIYSKFDLKSGFHQVAMHPESIPWTAFVVPPGQLYEWLVMPFGLKNAPAVFQRKMNQCFQGCENFV
2. NP_569153.1_CMBV TIFSKFDLKSGFHQVAMHPDSIEWTAFWVPSG-LYEWLVMPFGLKNAPAIFQRKMDHCFKGTEAFI
3. ACE76864.1_CMBV TIFSKFDLKSGFHQVAMHPDSVEWTAFWVPSG-LYEWLVMPFGLKNAPAVFQRKMDHCFKGTEAFI
4. YP_009130664.1_TaBV KIYSKFDLKSGFHQVAMHPYSIEWTAFWVPQG-LYEWLAMPFGLKNAPAVFQRKMDNCFKGTENFI
5. YP_009140788_GRBaV: KIFSKFDLKSGFHQVAMHPDSIEWTAFWVPDG-LYEWLVMPFGLKNAPSIFQRKMDECFNGTEEFI
6. YP_006273075.1_FBaV-1 KIFSKFDLKSGFHQVAMHPDSIEWTAFWVPDG-LYEWLVMPFGLKNAPSVFQRKMDECFKGTEDFI
7. ANK58542.1_FBaV-1 TIFSKFDLKSGFHQVAMHPDSIEWTAFWVPDG-LYEWLVMPFGLKNAPSVFQRKMDECFKGTEDFI
8. ATV81254.1_Grape RIYSKFDLKSGFHQVAMHPDSIEWTAFWVPDG-LYEWLVMPFGLKNAPAVFQRKMDHCFKGTEDFI
9. YP_001036293.1_DBSNV KIYSKFDLKSGFHQVAMAPQSVEWTAFLAPGG-LYEWLVMPFGLKNAPAVFQRKMDNVFRGTEEFI
10. ATW01249.1_DBSNV KIYSKFDLKSGFHQVAMAPQSIEWTAFLVPGG-LYEWLVMPFGLKNAPAVFQRKMDHVFRGTEDFI
11. AHG32725.1_DBSNV KIYSKFDLKSGFHQVAMAPQSIEWTAFLVPGG-LYEWLVMPFGLKNAPAVFQRKMDHVFRGTEDFI
12. YP_009002585.1_HBV KIYSKFDLKSGFHQVAMHPESIKWTAFWVPDG-LYEWLVMPFGLKNAPAVFQRKMDNVFKGTEAFI
13. YP_009113237.2_MBV-1 KIFSKFDLKSGFHQVAMHPESIPWTAFWVPQG-LYEWLVMPFGLKNAPAIFQRKMDNCFMGTEEFI
14. ATZ69526.1_CSSV KIYSKFDLKSGFHQIAMHPESIPWTAFWVPQG-LYEWLVMPFGIKNAPAIFQRKMDHCFAGMEEFI
15. ATZ69522.1_CSSV KIYSKFDLKSGFHQIAMHPESIPWTAFWVPQG-LYEWLVMPFGIKNAPAIFQRKMDHCFAGMEEFI
16. YP_003208050.1_PVBV KVYSKFDLKSGFHQVAMDEESIPWTAFCVPGG-LYEWLVMPFGLKNAPSVFQRKMDDCFKGTEAFI
17. YP_009229919.1_BVF KIYSKFDLKSGFHQVAMDEESIPWTAFITPEG-LYEWLVMPFGLKNAPAIFQRKMDNCFRGTEEFI
18. YP_009408594.1_Jujude KIYSKFDLKSGFHQIAMEKESIPWTAFIVPQG-LYEWLVMPFGLKNAPALFQRKMDNCFKGTEDFI
19. YP_610965.1_DrMV TVYSKFDLKSGFHQVAMSPESIEWTAFIVLGG-LYEWLVMPFGLKNAPAVFQRKMDHCFAGTEKFI
20. ABR01170.1_Lucky TVYSKFDLKSGFHQVAMSPESIEWTAFIVPGG-LYEWLVMPFG-KNAPAVFQRKMDHCFAGTEKFI
21. YP_009121747.1_YNMoV KIFSKFDLKSGFHQVAMEEESIPWTAFLIPGG-LYEWLVMPFGLKNAPAIFQRKMDKCFKDTEEFI
22. YP_009345075_CCYVB KIYSKFDLKSGFHQVAMEESSIPWTAFWVPDG-LYEWLVMPFGLKNAPAIFQRKMDNCFKGTEEFI
23. ASG91874.1_CCYVB KIFSKFDLKSGFHQVAMAEESIPWTAFWVPQG-LYEWLVMPFGLKNAPAVFQRKMDQCFKGTEEFI
24. AVT44083.1_CaYMV KIYSKFDLKSGFHQVAMEAESIPWTAFWAIDG-LYEWLVMPFGLKNAPAVFQRKMDSCFRGTEEFI
25. API68654.1_CaYMV KIYSKFDLKSGFHQVAMEAESIPWTAFWAIDG-LYEWLVMPFGLKNAPAVFQRKMDGCFKGTEEFI
26. YP_009259698.1_CaYMV RVYSKFDLKSGFHQVAMEEESIPWTAFWAIDG-LYEWLVMPFGLKNAPACFQRKMDNCFRGKEHFI
27. YP_003284237.1_ScBV KIYSKFDLKSGFHQVAMEEASIPWTAFWAIDG-LYEWLVMPFGLKNAPAVFQRKMDNCFRGTEEFI
28. YP_009116631.1_RYNV KIFSKFDLKSGFHQVLMDEESIPWTAFVTPVG-FYEWKVMPFGLANAPAVFQRKMDQCFAGTSEFI
29. AEE39279.1_GVBaV KIYSKFDLKAGFHQVLMEESSIPWTAFITPVG-FYEWLVMPFGIANAPAVFQRKMDNCFHKLREFV
30. AEE39276.1_GVBaV KIYSKFDLKAGFHQVLMEESSIPWTAFITPVG-FYEWLVMPFGIANAPAVFQRKMDNCFHKLREFV
31. AIY62341.1_GVCV KIFSKFDLKSGFHQVMMEEESIPWTAFISPAG-LYEWLVMPFGIQNAPAIFQRKMDECFKGTEDFI
32. AVA17853.1_BLRD KVFSKFDLKSGFHQVTMDEESIPWTAFLTPDG-LYEWLVMPFGLKNAPAVFQRKMDNCFKGTEEFI
33. YP_002321513.1_BsCVBV KIYSKFDLKSGFHQVAMDPESIPWTAFLTPQG-LFEWLVMPFGLKNAPAIFQRKMDNCFSRYSDFI
34. YP_009041481.1_PYMAV0 KVYSKFDLKSGFHQVAMDEASIPWTAFLVPGG-LYEWLVMPFGLRNAPAIFQRKMDEVFADLKEFV
35. YP_009352866.1_WBV KVYSKFDLKSGFHQVAMDEDSIPWTAFLVPGG-LYEWLVMPFGLRNAPAIFQRKMDEVFGDLKDFI

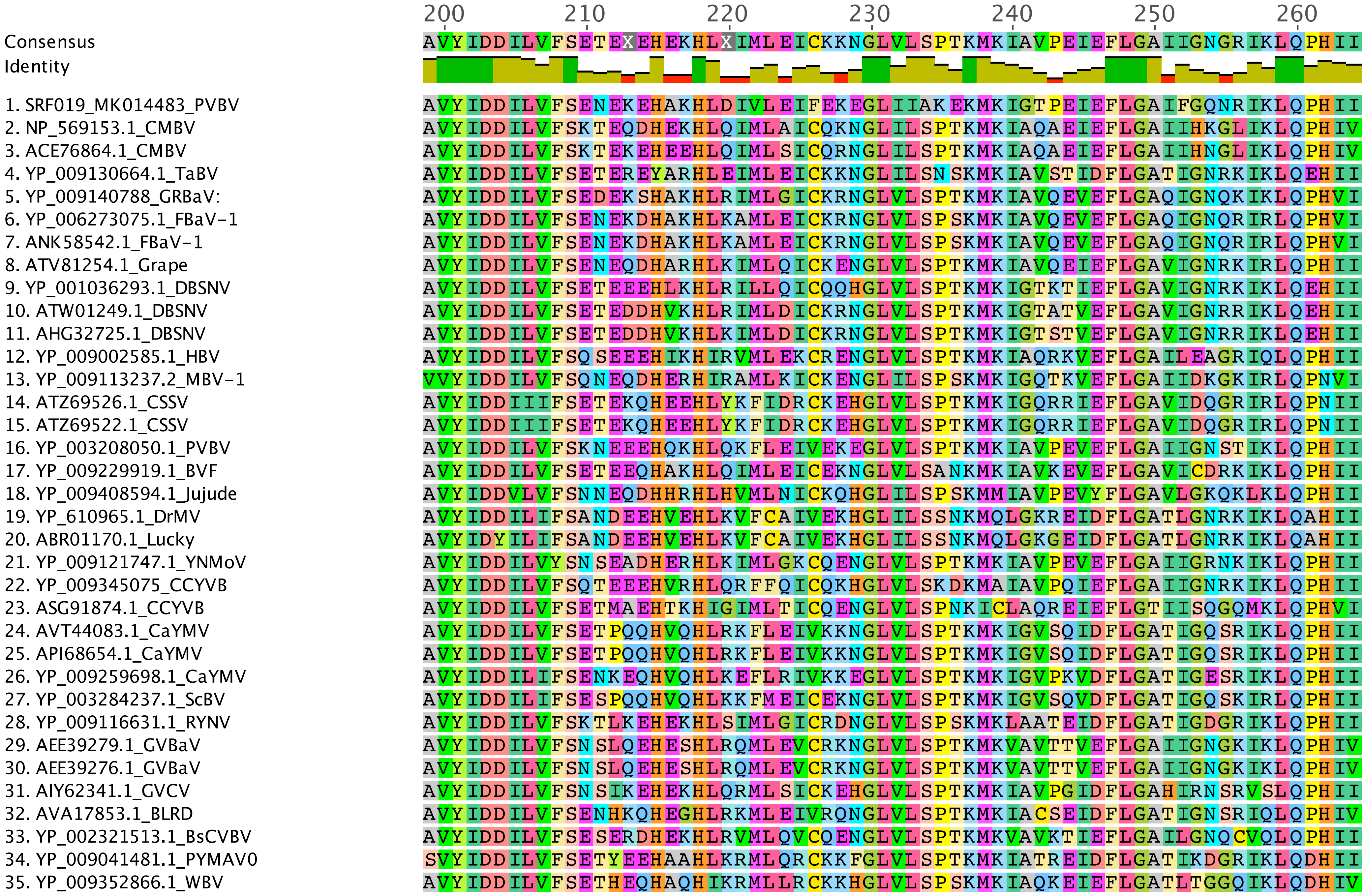
200 210 220 230 240 250 260
Consensus AVYIDDILVFSETEXEHEKHLXIMLEICKKNGLVLSPTKMKIAVPEIEFLGAIIGNGRIKLQPHII
Identity
1. SRF019_MK014483_PVBV AVYIDDILVFSENEKEHAKHLDIVLEIFEKEGLIIAKEKMKIGTPEIEFLGAIFGQNRIKLQPHII
2. NP_569153.1_CMBV AVYIDDILVFSKTEQDHEKHLQIMLAICQKNGLILSPTKMKIAQAEIEFLGAIIHKGLIKLQPHIV
3. ACE76864.1_CMBV AVYIDDILVFSKTEKEHEEHLQIMLSICQRNGLILSPTKMKIAQAEIEFLGAIIHNGLIKLQPHIV
4. YP_009130664.1_TaBV AVYIDDILVFSETEREYARHLEIMLEICKKNGLILSNSKMKIAVSTIDFLGATIGNRKIKLQEHII
5. YP_009140788_GRBaV: AVYIDDILVFSEDEKSHAKHLRIMLGICKRNGLVLSPTKMKIAVQEVEFLGAQIGNQKIKLQPHVI
6. YP_006273075.1_FBaV-1 AVYIDDILVFSENEKDHAKHLKAMLEICKRNGLVLSPSKMKIAVQEVEFLGAQIGNQRIRLQPHVI
7. ANK58542.1_FBaV-1 AVYIDDILVFSENEKDHAKHLKAMLEICKRNGLVLSPSKMKIAVQEVEFLGAQIGNQRIRLQPHVI
8. ATV81254.1_Grape AVYIDDILVFSENEQDHARHLKIMLQICKENGLVLSPTKMKIAVQEIEFLGAVIGNRKIRLQPHII
9. YP_001036293.1_DBSNV AVYIDDILVFSETEEEHLKHLRILLQICQQHGLVLSPTKMKIGTKTIEFLGAVIGNRKIKLQEHII
10. ATW01249.1_DBSNV AVYIDDILVFSETEDDHVKHLRIMLDICKRNGLVLSPTKMKIGTATVEFLGAVIGNRRIKLQEHII
11. AHG32725.1_DBSNV AVYIDDILVFSETEDDHVKHLKIMLDICKRNGLVLSPTKMKIGTSTVEFLGAVIGNRRIKLQEHII
12. YP_009002585.1_HBV AVYIDDILVFSQSEEEHIKHIRVMLEKCRENGLVLSPTKMKIAQRKVEFLGAILEAGRIQLQPHII
13. YP_009113237.2_MBV-1 VVYIDDILVFSQNEQDHERHIRAMLKICKENGLILSPSKMKIGQTKVEFLGAIIDKGKIRLQPNVI
14. ATZ69526.1_CSSV AVYIDDIIIFSETEKQHEEHLYKFIDRCKEHGLVLSPTKMKIGQRRIEFLGAVIDQGRIRLQPNII
15. ATZ69522.1_CSSV AVYIDDIIIFSETEKQHEEHLYKFIDRCKEHGLVLSPTKMKIGQRRIEFLGAVIDQGRIRLQPNII
16. YP_003208050.1_PVBV AVYIDDILVFSKNEEEHQKHLQKFLEIVEKEGLVLSPTKMKIAVPEVEFLGAIIGNSTIKLQPHII
17. YP_009229919.1_BVF AVYIDDILVFSETEEQHAKHLQIMLEICEKNGLVLSANKMKIAVKEVEFLGAVICDRKIKLQPHII
18. YP_009408594.1_Jujude AVYIDDVLVFSNNEQDHHRHLHVMLNICKQHGLILSPSKMMIAVPEVYFLGAVLGKQKLKLQPHII
19. YP_610965.1_DrMV AVYIDDILIFSANDEEHVEHLKVFCAIVEKHGLILSSNKMQLGKREIDFLGATLGNRKIKLQAHII
20. ABR01170.1_Lucky AVYIDYILIFSANDEEHVEHLKVFCAIVEKHGLILSSNKMQLGKGEIDFLGATLGNRKIKLQAHII
21. YP_009121747.1_YNMoV AVYIDDILVYSNSEADHERHLKIMLGKCQENGLVLSPTKMKIAVPEVEFLGAIIGRNKIKLQPHII
22. YP_009345075_CCYVB AVYIDDILVFSQTEEEHVRHLQRFFQICQKHGLVLSKDKMAIAVPQIEFLGAIIGNRKIKLQPHII
23. ASG91874.1_CCYVB AVYIDDILVFSETMAEHTKHIGIMLTICQENGLVLSPNKICLAQREIEFLGTIISQGQMKLQPHVI
24. AVT44083.1_CaYMV AVYIDDILVFSETPQQHVQHLRKFLEIVKKNGLVLSPTKMKIGVSQIDFLGATIGQSRIKLQPHII
25. API68654.1_CaYMV AVYIDDILVFSETPQQHVQHLRKFLEIVKKNGLVLSPTKMKIGVSQIDFLGATIGQSRIKLQPHII
26. YP_009259698.1_CaYMV AVYIDDILIFSENKEQHVQHLKEFLRIVKKEGLVLSPTKMKIGVPKVDFLGATIGESRIKLQPHII
27. YP_003284237.1_ScBV AVYIDDILIFSESPQQHVQHLKKFMEICEKNGLVLSPTKMKIGVSQVDFLGATIGQSKIRLQPHII
28. YP_009116631.1_RYNV AVYIDDILVFSKTLKEHEKHLSIMLGICRDNGLVLSPSKMKLAATEIDFLGATIGDGRIKLQPHII
29. AEE39279.1_GVBaV AVYIDDILVFSNSLQEHESHLRQMLEVCRKNGLVLSPTKMKVAVTTVEFLGAIIGNGKIKLQPHIV
30. AEE39276.1_GVBaV AVYIDDILVFSNSLQEHESHLRQMLEVCRKNGLVLSPTKMKVAVTTVEFLGAIIGNGKIKLQPHIV
31. AIY62341.1_GVCV AVYIDDILVFSNSIKEHEKHLQRMLSICKEHGLVLSPTKMKIAVPGIDFLGAHIRNSRVSLQPHII
32. AVA17853.1_BLRD AVYIDDILVFSENHKQHEGHLRKMLEIVRQNGLVLSPTKMKIACSEIDFLGATIGNSRIQLQPHIV
33. YP_002321513.1_BsCVBV AVYIDDILVFSESERDHEKHLRVMLQVCQENGLVLSPTKMKVAVKTIEFLGAILGNQCVQLQPHII
34. YP_009041481.1_PYMAV0 SVYIDDILVFSETYEEHAAHLKRMLQRCKKFGLVLSPTKMKIATREIDFLGATIKDGRIKLQDHII
35. YP_009352866.1_WBV AVYIDDILVFSETHEQHAQHIKRMLLRCKKHGLVLSPSKMKIAQKEIEFLGATLTGGQIKLQDHIV

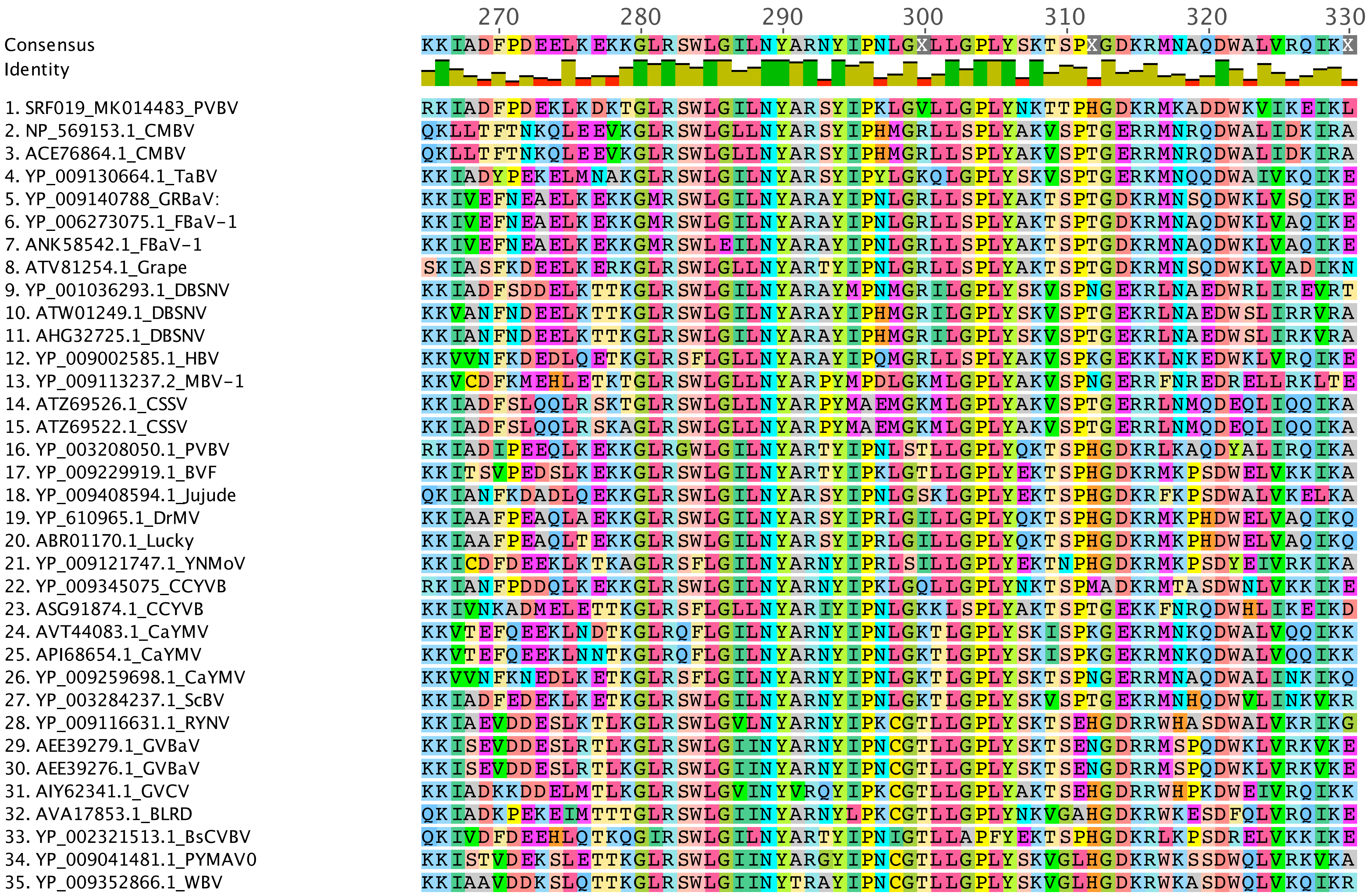
270 280 290 300 310 320 330
Consensus KKIADFPDEELKEKKGLRSWLGILNYARNYIPNLGXLLGPLYSKTSPXGDKRMNAQDWALVRQIKX
Identity
1. SRF019_MK014483_PVBV RKIADFPDEKLKDKTGLRSWLGILNYARSYIPKLGVLLGPLYNKTTPHGDKRMKADDWKVIKEIKL
2. NP_569153.1_CMBV QKLLTFTNKQLEEVKGLRSWLGLLNYARSYIPHMGRLLSPLYAKVSPTGERRMNRQDWALIDKIRA
3. ACE76864.1_CMBV QKLLTFTNKQLEEVKGLRSWLGLLNYARSYIPHMGRLLSPLYAKVSPTGERRMNRQDWALIDKIRA
4. YP_009130664.1_TaBV KKIADYPEKELMNAKGLRSWLGILNYARSYIPYLGKQLGPLYSKVSPTGERKMNQQDWAIVKQIKE
5. YP_009140788_GRBaV: KKIVEFNEAELKEKKGMRSWLGILNYARAYIPNLGRLLSPLYAKTSPTGDKRMNSQDWKLVSQIKE
6. YP_006273075.1_FBaV-1 KKIVEFNEAELKEKKGMRSWLGILNYARAYIPNLGRLLSPLYAKTSPTGDKRMNAQDWKLVAQIKE
7. ANK58542.1_FBaV-1 KKIVEFNEAELKEKKGMRSWLEILNYARAYIPNLGRLLSPLYAKTSPTGDKRMNAQDWKLVAQIKE
8. ATV81254.1_Grape SKIASFKDEELKERKGLRSWLGLLNYARTYIPNLGRLLSPLYAKTSPTGDKRMNSQDWKLVADIKN
9. YP_001036293.1_DBSNV KKIADFSDDELKTTKGLRSWLGILNYARAYMPNMGRILGPLYSKVSPNGEKRLNAEDWRLIREVRT
10. ATW01249.1_DBSNV KKVANFNDEELKTTKGLRSWLGILNYARAYIPHMGRILGPLYSKVSPTGEKRLNAEDWSLIRRVRA
11. AHG32725.1_DBSNV KKIANFNDEELKTTKGLRSWLGILNYARAYIPHMGRILGPLYSKVSPTGEKRLNAEDWSLIRKVRA
12. YP_009002585.1_HBV KKVVNFKDEDLQETKGLRSFLGLLNYARAYIPQMGRLLSPLYAKVSPKGEKKLNKEDWKLVRQIKE
13. YP_009113237.2_MBV-1 KKVCDFKMEHLETKTGLRSWLGLLNYARPYMPDLGKMLGPLYAKVSPNGERRFNREDRELLRKLTE
14. ATZ69526.1_CSSV KKIADFSLQQLRSKTGLRSWLGLLNYARPYMAEMGKMLGPLYAKVSPTGERRLNMQDEQLIQQIKA
15. ATZ69522.1_CSSV KKIADFSLQQLRSKAGLRSWLGLLNYARPYMAEMGKMLGPLYAKVSPTGERRLNMQDEQLIQQIKA
16. YP_003208050.1_PVBV RKIADIPEEQLKEKKGLRGWLGILNYARTYIPNLSTLLGPLYQKTSPHGDKRLKAQDYALIRQIKA
17. YP_009229919.1_BVF KKITSVPEDSLKEKKGLRSWLGILNYARTYIPKLGTLLGPLYEKTSPHGDKRMKPSDWELVKKIKA
18. YP_009408594.1_Jujude QKIANFKDADLQEKKGLRSWLGILNYARSYIPNLGSKLGPLYEKTSPHGDKRFKPSDWALVKELKA
19. YP_610965.1_DrMV KKIAAFPEAQLAEKKGLRSWLGILNYARSYIPRLGILLGPLYQKTSPHGDKRMKPHDWELVAQIKQ
20. ABR01170.1_Lucky KKIAAFPEAQLTEKKGLRSWLGILNYARSYIPRLGILLGPLYQKTSPHGDKRMKPHDWELVAQIKQ
21. YP_009121747.1_YNMoV KKICDFDEEKLKTKAGLRSFLGILNYARNYIPRLSILLGPLYEKTNPHGDKRMKPSDYEIVRKIKA
22. YP_009345075_CCYVB RKIANFPDDQLKEKKGLRSWLGILNYARNYIPKLGQLLGPLYNKTSPMADKRMTASDWNLVKKIKE
23. ASG91874.1_CCYVB KKIVNKADMELETTKGLRSFLGLLNYARIYIPNLGKKLSPLYAKTSPTGEKKFNRQDWHLIKEIKD
24. AVT44083.1_CaYMV KKVTEFQEEKLNDTKGLRQFLGILNYARNYIPNLGKTLGPLYSKISPKGEKRMNKQDWALVQQIKK
25. API68654.1_CaYMV KKVTEFQEEKLNNTKGLRQFLGILNYARNYIPNLGKTLGPLYSKISPKGEKRMNKQDWALVQQIKK
26. YP_009259698.1_CaYMV KKVVNFKNEDLKETKGLRSFLGILNYARNYIPNLGKTLGPLYSKTSPNGERRMNAQDWALINKIKQ
27. YP_003284237.1_ScBV KKIADFEDEKLKETKGLRSWLGILNYARNYIPNLGKTLGPLYSKVSPTGEKRMNHQDWVLINKVKR
28. YP_009116631.1_RYNV KKIAEVDDESLKTLKGLRSWLGVLNYARNYIPKCGTLLGPLYSKTSEHGDRRWHASDWALVKRIKG
29. AEE39279.1_GVBaV KKISEVDDESLRTLKGLRSWLGIINYARNYIPNCGTLLGPLYSKTSENGDRRMSPQDWKLVRKVKE
30. AEE39276.1_GVBaV KKISEVDDESLRTLKGLRSWLGIINYARNYIPNCGTLLGPLYSKTSENGDRRMSPQDWKLVRKVKE
31. AIY62341.1_GVCV KKIADKKDDELMTLKGLRSWLGVINYVRQYIPKCGTLLGPLYAKTSEHGDRRWHPKDWEIVRQIKK
32. AVA17853.1_BLRD QKIADKPEKEIMTTTGLRSWLGIINYARNYLPKCGTLLGPLYNKVGAHGDKRWKESDFQLVRQIKE
33. YP_002321513.1_BsCVBV QKIVDFDEEHLQTKQGIRSWLGILNYARTYIPNIGTLLAPFYEKTSPHGDKRLKPSDRELVKKIKE
34. YP_009041481.1_PYMAV0 KKISTVDEKSLETTKGLRSWLGIINYARGYIPNCGTLLGPLYSKVGLHGDKRWKSSDWQLVRKVKA
35. YP_009352866.1_WBV KKIAAVDDKSLQTTKGLRSWLGIINYTRAYIPNCGTLLGPLYSKVGLHGDKRWKASDWQLVKQIKR

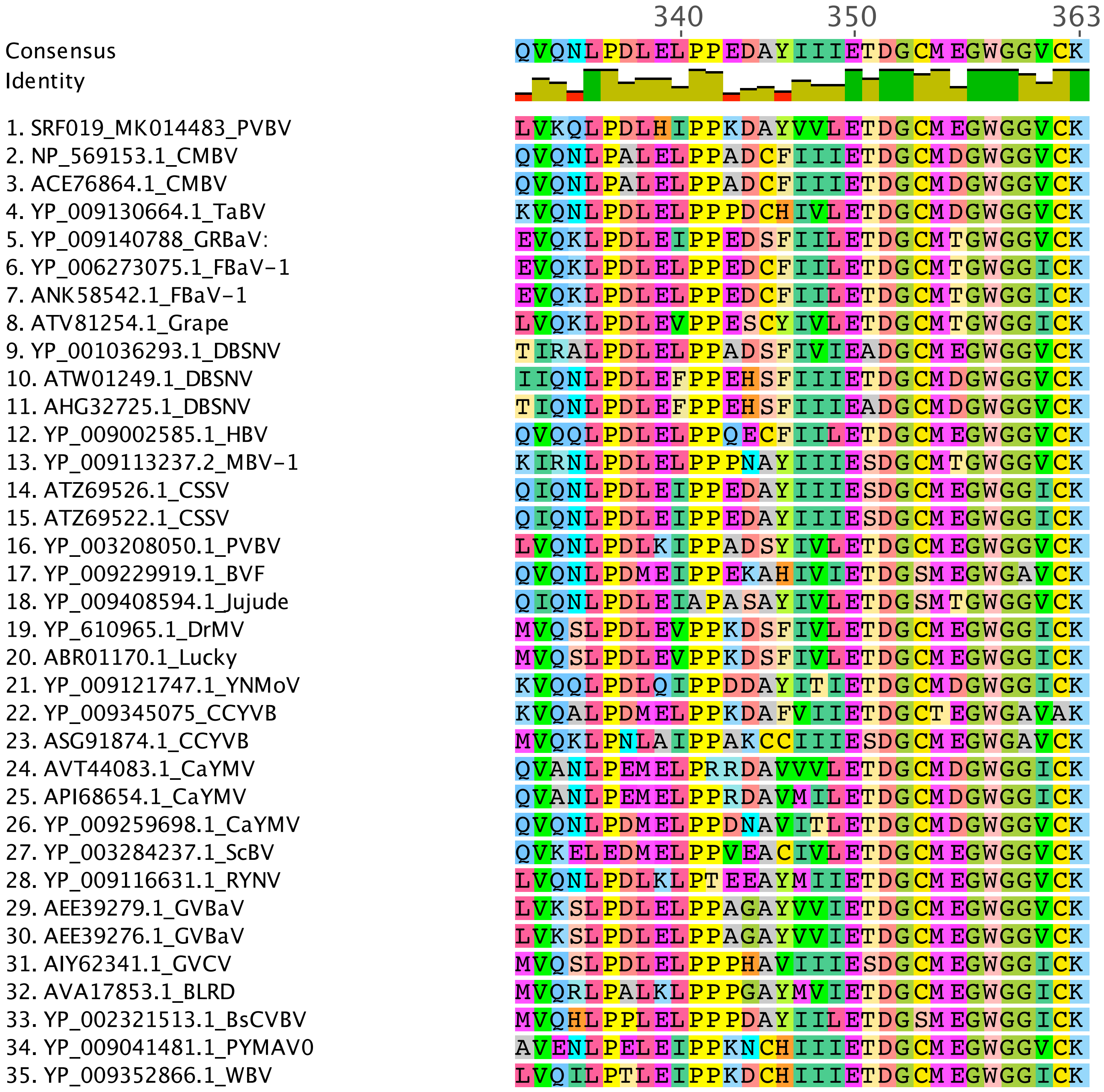

340
350
363
Consensus QVQNLPDLELPPEDAYIIIETDGCMEGWGGVCK
Identity
1. SRF019_MK014483_PVBV LVKQLPDLHIPPKDAYVVLETDGCMEGWGGVCK
2. NP_569153.1_CMBV QVQNLPALELPPADCFIIIETDGCMDGWGGVCK
3. ACE76864.1_CMBV QVQNLPALELPPADCFIIIETDGCMDGWGGVCK
4. YP_009130664.1_TaBV KVQNLPDLELPPPDCHIVLETDGCMDGWGGVCK
5. YP_009140788_GRBaV: EVQKLPDLEIPPEDSFIILETDGCMTGWGGVCK
6. YP_006273075.1_FBaV-1 EVQKLPDLELPPEDCFIILETDGCMTGWGGICK
7. ANK58542.1_FBaV-1 EVQKLPDLELPPEDCFIILETDGCMTGWGGICK
8. ATV81254.1_Grape LVQKLPDLEVPPESCYIVLETDGCMTGWGGICK
9. YP_001036293.1_DBSNV TIRALPDLELPPADSFIVIEADGCMEGWGGVCK
10. ATW01249.1_DBSNV IIQNLPDLEFPPEHSFIIIETDGCMDGWGGVCK
11. AHG32725.1_DBSNV TIQNLPDLEFPPEHSFIIIEADGCMDGWGGVCK
12. YP_009002585.1_HBV QVQQLPDLELPPQECFIILETDGCMEGWGGVCK
13. YP_009113237.2_MBV-1 KIRNLPDLELPPPNAYIIIESDGCMTGWGGVCK
14. ATZ69526.1_CSSV QIQNLPDLEIPPEDAYIIIESDGCMEGWGGICK
15. ATZ69522.1_CSSV QIQNLPDLEIPPEDAYIIIESDGCMEGWGGICK
16. YP_003208050.1_PVBV LVQNLPDLKIPPADSYIVLETDGCMEGWGGVCK
17. YP_009229919.1_BVF QVQNLPDMEIPPEKAHIVIETDGSMEGWGAVCK
18. YP_009408594.1_Jujude QIQNLPDLEIAPASAYIVLETDGSMTGWGGVCK
19. YP_610965.1_DrMV MVQSLPDLEVPPKDSFIVLETDGCMEGWGGICK
20. ABR01170.1_Lucky MVQSLPDLEVPPKDSFIVLETDGCMEGWGGICK
21. YP_009121747.1_YNMoV KVQQLPDLQIPPDDAYITIETDGCMDGWGGICK
22. YP_009345075_CCYVB KVQALPDMELPPKDAFVIIETDGCTEGWGAVAK
23. ASG91874.1_CCYVB MVQKLPNLAIPPAKCCIIIESDGCMEGWGAVCK
24. AVT44083.1_CaYMV QVANLPEMELPRRDAVVVLETDGCMDGWGGICK
25. API68654.1_CaYMV QVANLPEMELPPRDAVMILETDGCMDGWGGICK
26. YP_009259698.1_CaYMV QVQNLPDMELPPDNAVITLETDGCMDGWGGVCK
27. YP_003284237.1_ScBV QVKELEDMELPPVEACIVLETDGCMEGWGGVCK
28. YP_009116631.1_RYNV LVQNLPDLKLPTEEAYMIIETDGCMEGWGGVCK
29. AEE39279.1_GVBaV LVKSLPDLELPPAGAYVVIETDGCMEGWGGVCK
30. AEE39276.1_GVBaV LVKSLPDLELPPAGAYVVIETDGCMEGWGGVCK
31. AIY62341.1_GVCV MVQSLPDLELPPPHAVIIIESDGCMEGWGGICK
32. AVA17853.1_BLRD MVQRLPALKLPPPGAYMVIETDGCMEGWGGICK
33. YP_002321513.1_BsCVBV MVQHLPPLELPPPDAYIILETDGSMEGWGGICK
34. YP_009041481.1_PYMAV0 AVENLPELEIPPKNCHIIIETDGCMEGWGGVCK
35. YP_009352866.1_WBV LVQILPTLEIPPKDCHIIIETDGCMEGWGGICK
