# Supplementary material for: A metagenomic study of DNA viruses from samples of local varieties of common bean in Kenya
Source: PeerJ. 2019 Mar 15;7:e6465. doi: 10.7717/peerj.6465 (PMC6422016; doi:10.7717/peerj.6465)
Supplement: Supplemental Information 6 — Sequence alignment of the amino acid sequences of the RdRp region of Bean common mosaic necrosis virus using MAFFT (Katoh & Standley, 2016). [file peerj-07-6465-s006.pdf]

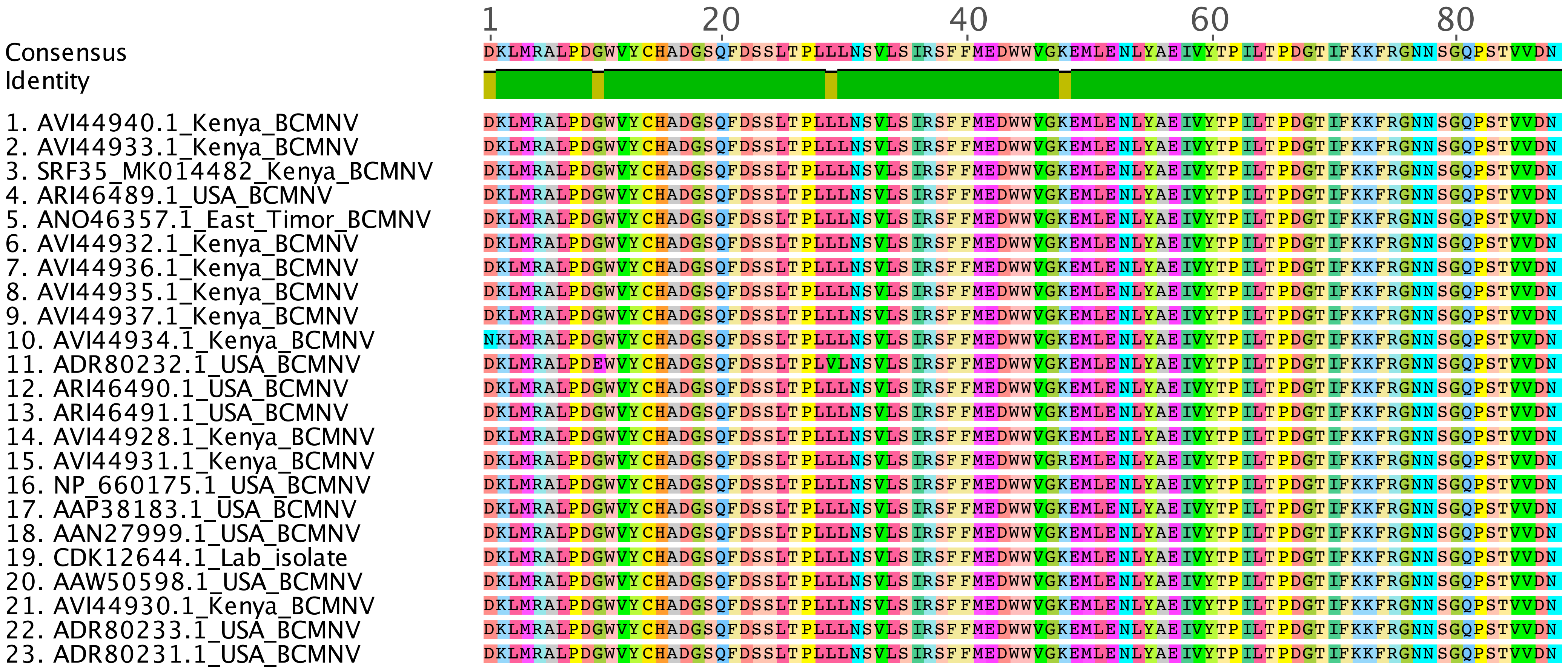

1
20
40
60
80
Consensus DKLMRALPDGWVYCHADGSQFDSSLTPLLLNSVLSIRSFFMEDWWVGKEMLENLYAEIVYTPILTPDGTIFKKFRGNNSGQPSTVVDN
Identity
1. AVI44940.1_Kenya_BCMNV DKLMRALPDGWVYCHADGSQFDSSLTPLLLNSVLSIRSFFMEDWWVGKEMLENLYAEIVYTPILTPDGTIFKKFRGNNSGQPSTVVDN
2. AVI44933.1_Kenya_BCMNV DKLMRALPDGWVYCHADGSQFDSSLTPLLLNSVLSIRSFFMEDWWVGKEMLENLYAEIVYTPILTPDGTIFKKFRGNNSGQPSTVVDN
3. SRF35_MK014482_Kenya_BCMNV DKLMRALPDGWVYCHADGSQFDSSLTPLLLNSVLSIRSFFMEDWWVGKEMLENLYAEIVYTPILTPDGTIFKKFRGNNSGQPSTVVDN
4. ARI46489.1_USA_BCMNV DKLMRALPDGWVYCHADGSQFDSSLTPLLLNSVLSIRSFFMEDWWVGKEMLENLYAEIVYTPILTPDGTIFKKFRGNNSGQPSTVVDN
5. ANO46357.1_East_Timor_BCMNV DKLMRALPDGWVYCHADGSQFDSSLTPLLLNSVLSIRSFFMEDWWVGKEMLENLYAEIVYTPILTPDGTIFKKFRGNNSGQPSTVVDN
6. AVI44932.1_Kenya_BCMNV DKLMRALPDGWVYCHADGSQFDSSLTPLLLNSVLSIRSFFMEDWWVGKEMLENLYAEIVYTPILTPDGTIFKKFRGNNSGQPSTVVDN
7. AVI44936.1_Kenya_BCMNV DKLMRALPDGWVYCHADGSQFDSSLTPLLLNSVLSIRSFFMEDWWVGKEMLENLYAEIVYTPILTPDGTIFKKFRGNNSGQPSTVVDN
8. AVI44935.1_Kenya_BCMNV DKLMRALPDGWVYCHADGSQFDSSLTPLLLNSVLSIRSFFMEDWWVGKEMLENLYAEIVYTPILTPDGTIFKKFRGNNSGQPSTVVDN
9. AVI44937.1_Kenya_BCMNV DKLMRALPDGWVYCHADGSQFDSSLTPLLLNSVLSIRSFFMEDWWVGKEMLENLYAEIVYTPILTPDGTIFKKFRGNNSGQPSTVVDN
10. AVI44934.1_Kenya_BCMNV NKLMRALPDGWVYCHADGSQFDSSLTPLLLNSVLSIRSFFMEDWWVGKEMLENLYAEIVYTPILTPDGTIFKKFRGNNSGQPSTVVDN
11. ADR80232.1_USA_BCMNV DKLMRALPDEWVYCHADGSQFDSSLTPLVLNSVLSIRSFFMEDWWVGKEMLENLYAEIVYTPILTPDGTIFKKFRGNNSGQPSTVVDN
12. ARI46490.1_USA_BCMNV DKLMRALPDGWVYCHADGSQFDSSLTPLLLNSVLSIRSFFMEDWWVGKEMLENLYAEIVYTPILTPDGTIFKKFRGNNSGQPSTVVDN
13. ARI46491.1_USA_BCMNV DKLMRALPDGWVYCHADGSQFDSSLTPLLLNSVLSIRSFFMEDWWVGKEMLENLYAEIVYTPILTPDGTIFKKFRGNNSGQPSTVVDN
14. AVI44928.1_Kenya_BCMNV DKLMRALPDGWVYCHADGSQFDSSLTPLLLNSVLSIRSFFMEDWWVGKEMLENLYAEIVYTPILTPDGTIFKKFRGNNSGQPSTVVDN
15. AVI44931.1_Kenya_BCMNV DKLMRALPDGWVYCHADGSQFDSSLTPLLLNSVLSIRSFFMEDWWVGREMLENLYAEIVYTPILTPDGTIFKKFRGNNSGQPSTVVDN
16. NP_660175.1_USA_BCMNV DKLMRALPDGWVYCHADGSQFDSSLTPLLLNSVLSIRSFFMEDWWVGKEMLENLYAEIVYTPILTPDGTIFKKFRGNNSGQPSTVVDN
17. AAP38183.1_USA_BCMNV DKLMRALPDGWVYCHADGSQFDSSLTPLLLNSVLSIRSFFMEDWWVGKEMLENLYAEIVYTPILTPDGTIFKKFRGNNSGQPSTVVDN
18. AAN27999.1_USA_BCMNV DKLMRALPDGWVYCHADGSQFDSSLTPLLLNSVLSIRSFFMEDWWVGKEMLENLYAEIVYTPILTPDGTIFKKFRGNNSGQPSTVVDN
19. CDK12644.1_Lab_isolate DKLMRALPDGWVYCHADGSQFDSSLTPLLLNSVLSIRSFFMEDWWVGKEMLENLYAEIVYTPILTPDGTIFKKFRGNNSGQPSTVVDN
20. AAW50598.1_USA_BCMNV DKLMRALPDGWVYCHADGSQFDSSLTPLLLNSVLSIRSFFMEDWWVGKEMLENLYAEIVYTPILTPDGTIFKKFRGNNSGQPSTVVDN
21. AVI44930.1_Kenya_BCMNV DKLMRALPDGWVYCHADGSQFDSSLTPLLLNSVLSIRSFFMEDWWVGKEMLENLYAEIVYTPILTPDGTIFKKFRGNNSGQPSTVVDN
22. ADR80233.1_USA_BCMNV DKLMRALPDGWVYCHADGSQFDSSLTPLLLNSVLSIRSFFMEDWWVGKEMLENLYAEIVYTPILTPDGTIFKKFRGNNSGQPSTVVDN
23. ADR80231.1_USA_BCMNV DKLMRALPDGWVYCHADGSQFDSSLTPLLLNSVLSIRSFFMEDWWVGKEMLENLYAEIVYTPILTPDGTIFKKFRGNNSGQPSTVVDN

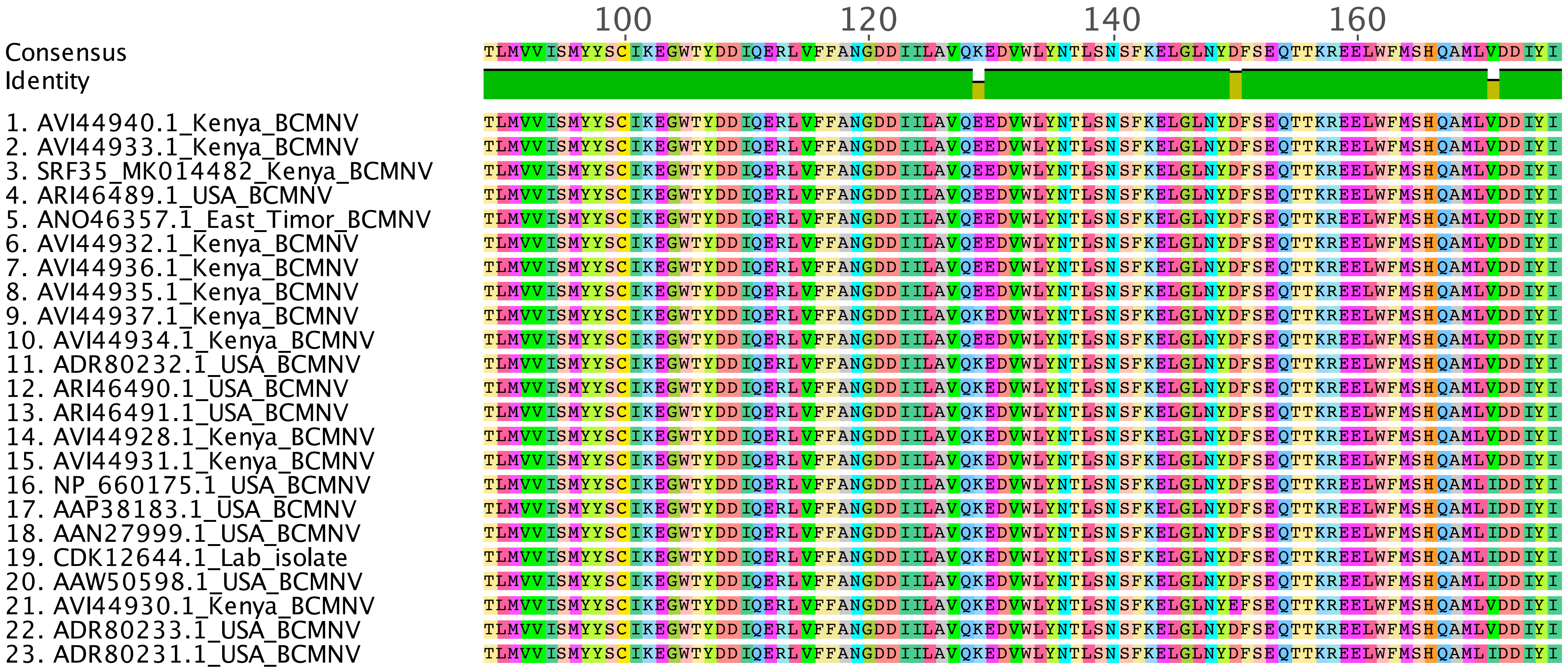

100
120
140
160
Consensus TLMVVISMYYSCIKEGWTYDDIQERLVFFANGDDIILAVQKEDVWLYNTLSNSFKELGLNYDFSEQTTKREELWFMSHQAMLVDDIYI
Identity
1. AVI44940.1_Kenya_BCMNV TLMVVISMYYSCIKEGWTYDDIQERLVFFANGDDIILAVQEEDVWLYNTLSNSFKELGLNYDFSEQTTKREELWFMSHQAMLVDDIYI
2. AVI44933.1_Kenya_BCMNV TLMVVISMYYSCIKEGWTYDDIQERLVFFANGDDIILAVQEEDVWLYNTLSNSFKELGLNYDFSEQTTKREELWFMSHQAMLVDDIYI
3. SRF35_MK014482_Kenya_BCMNV TLMVVISMYYSCIKEGWTYDDIQERLVFFANGDDIILAVQEEDVWLYNTLSNSFKELGLNYDFSEQTTKREELWFMSHQAMLVDDIYI
4. ARI46489.1_USA_BCMNV TLMVVISMYYSCIKEGWTYDDIQERLVFFANGDDIILAVQEEDVWLYNTLSNSFKELGLNYDFSEQTTKREELWFMSHQAMLVDDIYI
5. ANO46357.1_East_Timor_BCMNV TLMVVISMYYSCIKEGWTYDDIQERLVFFANGDDIILAVQEEDVWLYNTLSNSFKELGLNYDFSEQTTKREELWFMSHQAMLVDDIYI
6. AVI44932.1_Kenya_BCMNV TLMVVISMYYSCIKEGWTYDDIQERLVFFANGDDIILAVQEEDVWLYNTLSNSFKELGLNYDFSEQTTKREELWFMSHQAMLVDDIYI
7. AVI44936.1_Kenya_BCMNV TLMVVISMYYSCIKEGWTYDDIQERLVFFANGDDIILAVQEEDVWLYNTLSNSFKELGLNYDFSEQTTKREELWFMSHQAMLVDDIYI
8. AVI44935.1_Kenya_BCMNV TLMVVISMYYSCIKEGWTYDDIQERLVFFANGDDIILAVQEEDVWLYNTLSNSFKELGLNYDFSEQTTKREELWFMSHQAMLVDDIYI
9. AVI44937.1_Kenya_BCMNV TLMVVISMYYSCIKEGWTYDDIQERLVFFANGDDIILAVQKEDVWLYNTLSNSFKELGLNYDFSEQTTKREELWFMSHQAMLVDDIYI
10. AVI44934.1_Kenya_BCMNV TLMVVISMYYSCIKEGWTYDDIQERLVFFANGDDIILAVQEEDVWLYNTLSNSFKELGLNYDFSEQTTKREELWFMSHQAMLVDDIYI
11. ADR80232.1_USA_BCMNV TLMVVISMYYSCIKEGWTYDDIQERLVFFANGDDIILAVQKEDVWLYNTLSNSFKELGLNYDFSEQTTKREELWFMSHQAMLVDDIYI
12. ARI46490.1_USA_BCMNV TLMVVISMYYSCIKEGWTYDDIQERLVFFANGDDIILAVQKEDVWLYNTLSNSFKELGLNYDFSEQTTKREELWFMSHQAMLVDDIYI
13. ARI46491.1_USA_BCMNV TLMVVISMYYSCIKEGWTYDDIQERLVFFANGDDIILAVQKEDVWLYNTLSNSFKELGLNYDFSEQTTKREELWFMSHQAMLVDDIYI
14. AVI44928.1_Kenya_BCMNV TLMVVISMYYSCIKEGWTYDDIQERLVFFANGDDIILAVQKEDVWLYNTLSNSFKELGLNYDFSEQTTKREELWFMSHQAMLVDDIYI
15. AVI44931.1_Kenya_BCMNV TLMVVISMYYSCIKEGWTYDDIQERLVFFANGDDIILAVQKEDVWLYNTLSNSFKELGLNYDFSEQTTKREELWFMSHQAMLVDDIYI
16. NP_660175.1_USA_BCMNV TLMVVISMYYSCIKEGWTYDDIQERLVFFANGDDIILAVQKEDVWLYNTLSNSFKELGLNYDFSEQTTKREELWFMSHQAMLIDDIYI
17. AAP38183.1_USA_BCMNV TLMVVISMYYSCIKEGWTYDDIQERLVFFANGDDIILAVQKEDVWLYNTLSNSFKELGLNYDFSEQTTKREELWFMSHQAMLIDDIYI
18. AAN27999.1_USA_BCMNV TLMVVISMYYSCIKEGWTYDDIQERLVFFANGDDIILAVQKEDVWLYNTLSNSFKELGLNYDFSEQTTKREELWFMSHQAMLIDDIYI
19. CDK12644.1_Lab_isolate TLMVVISMYYSCIKEGWTYDDIQERLVFFANGDDIILAVQKEDVWLYNTLSNSFKELGLNYDFSEQTTKREELWFMSHQAMLIDDIYI
20. AAW50598.1_USA_BCMNV TLMVVISMYYSCIKEGWTYDDIQERLVFFANGDDIILAVQKEDVWLYNTLSNSFKELGLNYDFSEQTTKREELWFMSHQAMLIDDIYI
21. AVI44930.1_Kenya_BCMNV TLMVVISMYYSCIKEGWTYDDIQERLVFFANGDDIILAVQKEDVWLYNTLSNSFKELGLNYEFSEQTTKREELWFMSHQAMLVDDIYI
22. ADR80233.1_USA_BCMNV TLMVVISMYYSCIKEGWTYDDIQERLVFFANGDDIILAVQKEDVWLYNTLSNSFKELGLNYDFSEQTTKREELWFMSHQAMLVDDIYI
23. ADR80231.1_USA_BCMNV TLMVVISMYYSCIKEGWTYDDIQERLVFFANGDDIILAVQKEDVWLYNTLSNSFKELGLNYDFSEQTTKREELWFMSHQAMLIDDIYI

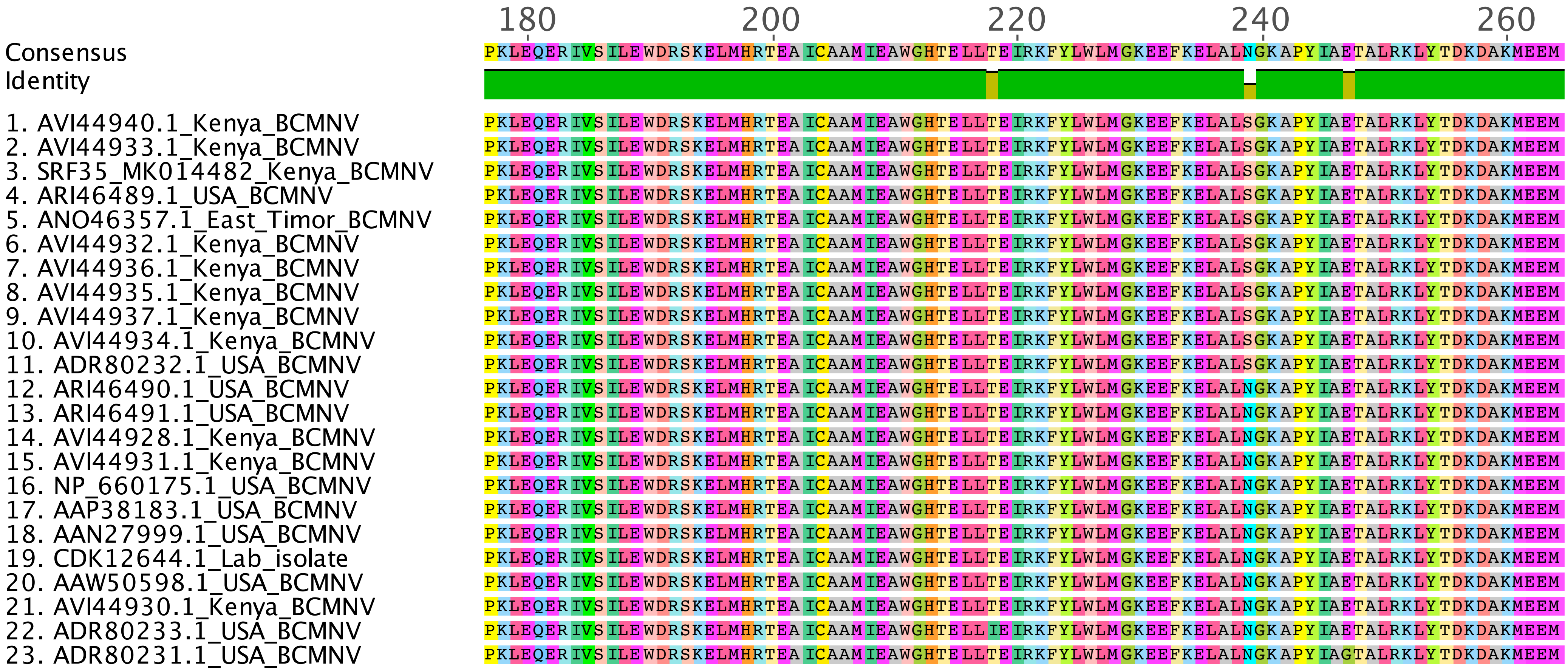

180
200
220
240
260
Consensus
PKLEQERIVSILEWDRSKELMHRTEAICAAMIEAWGHTELLTEIRKFYLWLMGKEEFKELALNGKAPYIAETALRKLYTDKDAKMEEM
Identity
1. AVI44940.1_Kenya_BCMNV
PKLEQERIVSILEWDRSKELMHRTEAICAAMIEAWGHTELLTEIRKFYLWLMGKEEFKELALSGKAPYIAETALRKLYTDKDAKMEEM
2. AVI44933.1_Kenya_BCMNV
PKLEQERIVSILEWDRSKELMHRTEAICAAMIEAWGHTELLTEIRKFYLWLMGKEEFKELALSGKAPYIAETALRKLYTDKDAKMEEM
3. SRF35_MK014482_Kenya_BCMNV
PKLEQERIVSILEWDRSKELMHRTEAICAAMIEAWGHTELLTEIRKFYLWLMGKEEFKELALSGKAPYIAETALRKLYTDKDAKMEEM
4. ARI46489.1_USA_BCMNV
PKLEQERIVSILEWDRSKELMHRTEAICAAMIEAWGHTELLTEIRKFYLWLMGKEEFKELALSGKAPYIAETALRKLYTDKDAKMEEM
5. ANO46357.1_East_Timor_BCMNV
PKLEQERIVSILEWDRSKELMHRTEAICAAMIEAWGHTELLTEIRKFYLWLMGKEEFKELALSGKAPYIAETALRKLYTDKDAKMEEM
6. AVI44932.1_Kenya_BCMNV
PKLEQERIVSILEWDRSKELMHRTEAICAAMIEAWGHTELLTEIRKFYLWLMGKEEFKELALSGKAPYIAETALRKLYTDKDAKMEEM
7. AVI44936.1_Kenya_BCMNV
PKLEQERIVSILEWDRSKELMHRTEAICAAMIEAWGHTELLTEIRKFYLWLMGKEEFKELALSGKAPYIAETALRKLYTDKDAKMEEM
8. AVI44935.1_Kenya_BCMNV
PKLEQERIVSILEWDRSKELMHRTEAICAAMIEAWGHTELLTEIRKFYLWLMGKEEFKELALSGKAPYIAETALRKLYTDKDAKMEEM
9. AVI44937.1_Kenya_BCMNV
PKLEQERIVSILEWDRSKELMHRTEAICAAMIEAWGHTELLTEIRKFYLWLMGKEEFKELALSGKAPYIAETALRKLYTDKDAKMEEM
10. AVI44934.1_Kenya_BCMNV
PKLEQERIVSILEWDRSKELMHRTEAICAAMIEAWGHTELLTEIRKFYLWLMGKEEFKELALSGKAPYIAETALRKLYTDKDAKMEEM
11. ADR80232.1_USA_BCMNV
PKLEQERIVSILEWDRSKELMHRTEAICAAMIEAWGHTELLTEIRKFYLWLMGKEEFKELALSGKAPYIAETALRKLYTDKDAKMEEM
12. ARI46490.1_USA_BCMNV
PKLEQERIVSILEWDRSKELMHRTEAICAAMIEAWGHTELLTEIRKFYLWLMGKEEFKELALNGKAPYIAETALRKLYTDKDAKMEEM
13. ARI46491.1_USA_BCMNV
PKLEQERIVSILEWDRSKELMHRTEAICAAMIEAWGHTELLTEIRKFYLWLMGKEEFKELALNGKAPYIAETALRKLYTDKDAKMEEM
14. AVI44928.1_Kenya_BCMNV
PKLEQERIVSILEWDRSKELMHRTEAICAAMIEAWGHTELLTEIRKFYLWLMGKEEFKELALNGKAPYIAETALRKLYTDKDAKMEEM
15. AVI44931.1_Kenya_BCMNV
PKLEQERIVSILEWDRSKELMHRTEAICAAMIEAWGHTELLTEIRKFYLWLMGKEEFKELALNGKAPYIAETALRKLYTDKDAKMEEM
16. NP_660175.1_USA_BCMNV
PKLEQERIVSILEWDRSKELMHRTEAICAAMIEAWGHTELLTEIRKFYLWLMGKEEFKELALNGKAPYIAETALRKLYTDKDAKMEEM
17. AAP38183.1_USA_BCMNV
PKLEQERIVSILEWDRSKELMHRTEAICAAMIEAWGHTELLTEIRKFYLWLMGKEEFKELALNGKAPYIAETALRKLYTDKDAKMEEM
18. AAN27999.1_USA_BCMNV
PKLEQERIVSILEWDRSKELMHRTEAICAAMIEAWGHTELLTEIRKFYLWLMGKEEFKELALNGKAPYIAETALRKLYTDKDAKMEEM
19. CDK12644.1_Lab_isolate
PKLEQERIVSILEWDRSKELMHRTEAICAAMIEAWGHTELLTEIRKFYLWLMGKEEFKELALNGKAPYIAETALRKLYTDKDAKMEEM
20. AAW50598.1_USA_BCMNV
PKLEQERIVSILEWDRSKELMHRTEAICAAMIEAWGHTELLTEIRKFYLWLMGKEEFKELALNGKAPYIAETALRKLYTDKDAKMEEM
21. AVI44930.1_Kenya_BCMNV
PKLEQERIVSILEWDRSKELMHRTEAICAAMIEAWGHTELLTEIRKFYLWLMGKEEFKELALNGKAPYIAETALRKLYTDKDAKMEEM
22. ADR80233.1_USA_BCMNV
PKLEQERIVSILEWDRSKELMHRTEAICAAMIEAWGHTELLIEIRKFYLWLMGKEEFKELALNGKAPYIAETALRKLYTDKDAKMEEM
23. ADR80231.1_USA_BCMNV
PKLEQERIVSILEWDRSKELMHRTEAICAAMIEAWGHTELLTEIRKFYLWLMGKEEFKELALNGKAPYIAGTALRKLYTDKDAKMEEM

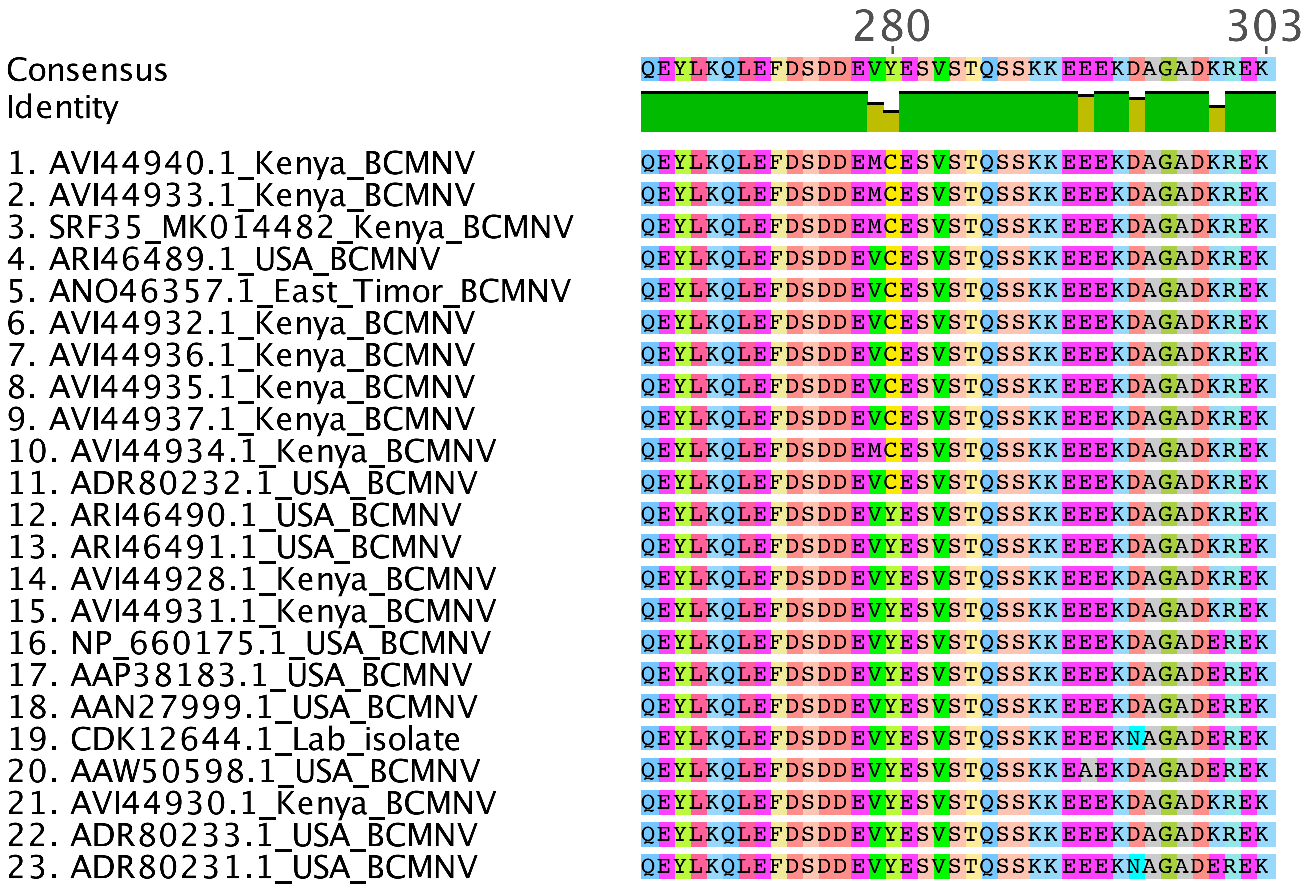
280
303
Consensus
Identity
QEYLKQLEFDSDDEVYESVSTQSSKKEEEKDAGADKREK
1. AVI44940.1_Kenya_BCMNV QEYLKQLEFDSDDEMCESVSTQSSKKEEEKDAGADKREK
2. AVI44933.1_Kenya_BCMNV QEYLKQLEFDSDDEMCESVSTQSSKKEEEKDAGADKREK
3. SRF35_MK014482_Kenya_BCMNV QEYLKQLEFDSDDEMCESVSTQSSKKEEEKDAGADKREK
4. ARI46489.1_USA_BCMNV QEYLKQLEFDSDDEVCESVSTQSSKKEEEKDAGADKREK
5. ANO46357.1_East_Timor_BCMNV QEYLKQLEFDSDDEVCESVSTQSSKKEEEKDAGADKREK
6. AVI44932.1_Kenya_BCMNV QEYLKQLEFDSDDEVCESVSTQSSKKEEEKDAGADKREK
7. AVI44936.1_Kenya_BCMNV QEYLKQLEFDSDDEVCESVSTQSSKKEEEKDAGADKREK
8. AVI44935.1_Kenya_BCMNV QEYLKQLEFDSDDEVCESVSTQSSKKEEEKDAGADKREK
9. AVI44937.1_Kenya_BCMNV QEYLKQLEFDSDDEVCESVSTQSSKKEEEKDAGADKREK
10. AVI44934.1_Kenya_BCMNV QEYLKQLEFDSDDEMCESVSTQSSKKEEEKDAGADKREK
11. ADR80232.1_USA_BCMNV QEYLKQLEFDSDDEVCESVSTQSSKKEEEKDAGADKREK
12. ARI46490.1_USA_BCMNV QEYLKQLEFDSDDEVYESVSTQSSKKEEEKDAGADKREK
13. ARI46491.1_USA_BCMNV QEYLKQLEFDSDDEVYESVSTQSSKKEEEKDAGADKREK
14. AVI44928.1_Kenya_BCMNV QEYLKQLEFDSDDEVYESVSTQSSKKEEEKDAGADKREK
15. AVI44931.1_Kenya_BCMNV QEYLKQLEFDSDDEVYESVSTQSSKKEEEKDAGADKREK
16. NP_660175.1_USA_BCMNV QEYLKQLEFDSDDEVYESVSTQSSKKEEEKDAGADEREK
17. AAP38183.1_USA_BCMNV QEYLKQLEFDSDDEVYESVSTQSSKKEEEKDAGADEREK
18. AAN27999.1_USA_BCMNV QEYLKQLEFDSDDEVYESVSTQSSKKEEEKDAGADEREK
19. CDK12644.1_Lab_isolate QEYLKQLEFDSDDEVYESVSTQSSKKEEEKNAGADEREK
20. AAW50598.1_USA_BCMNV QEYLKQLEFDSDDEVYESVSTQSSKKEAEKDAGADEREK
21. AVI44930.1_Kenya_BCMNV QEYLKQLEFDSDDEVYESVSTQSSKKEEEKDAGADKREK
22. ADR80233.1_USA_BCMNV QEYLKQLEFDSDDEVYESVSTQSSKKEEEKDAGADKREK
23. ADR80231.1_USA_BCMNV QEYLKQLEFDSDDEVYESVSTQSSKKEEEKNAGADEREK
