# Supplementary material for: A metagenomic study of DNA viruses from samples of local varieties of common bean in Kenya
Source: PeerJ. 2019 Mar 15;7:e6465. doi: 10.7717/peerj.6465 (PMC6422016; doi:10.7717/peerj.6465)
Supplement: Supplemental Information 7 — Sequence alignment nucleotide sequences of the hypothetical protein 1 and 2 region of the Aphid lethal paralysis virus using MAFFT (Katoh & Standley, 2016). [file peerj-07-6465-s007.pdf]

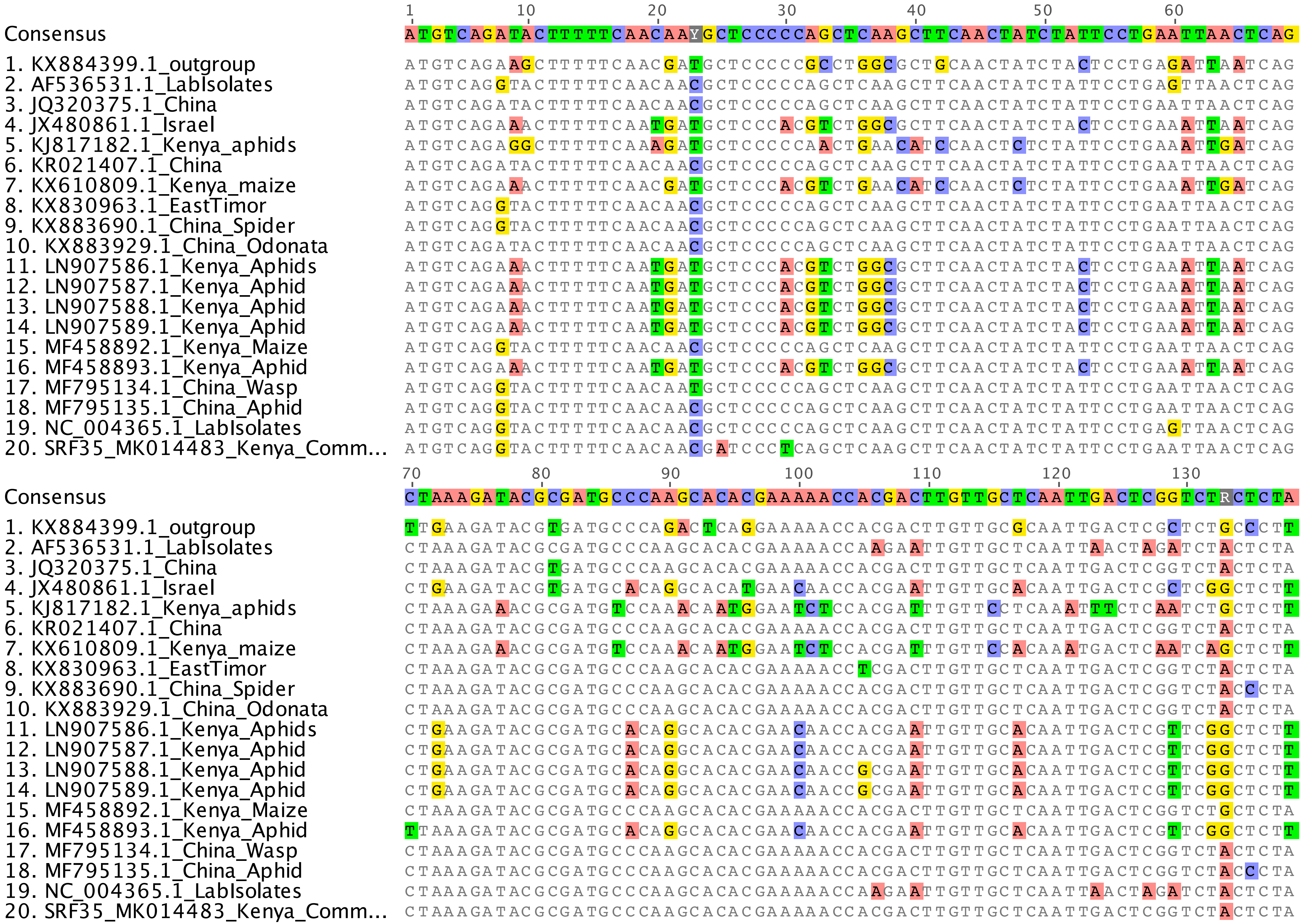
1 10 20 30 40 50 60
Consensus ATGTCAGATACTTTTTCAACAAYGCTCCCCCAGCTCAAGCTTCAACTATCTATTCCTGAATTAACTCAG
1. KX884399.1_outgroup ATGTCAGAAGCTTTTTCAACGATGCTCCCCCGCCTGGCGCTGCAACTATCTACTCCTGAGATTAATCAG
2. AF536531.1_LabIsolates ATGTCAGGTACTTTTTCAACAACGCTCCCCCAGCTCAAGCTTCAACTATCTATTCCTGAGTTAACTCAG
3. JQ320375.1_China ATGTCAGATACTTTTTCAACAACGCTCCCCCAGCTCAAGCTTCAACTATCTATTCCTGAATTAACTCAG
4. JX480861.1_Israel ATGTCAGAAACTTTTTCAATGATGCTCCCACGTCTGGCGCTTCAACTATCTACTCCTGAAATTAATCAG
5. KJ817182.1_Kenya_aphids ATGTCAGAGGCTTTTTCAAAGATGCTCCCCCAACTGAACATCCAACTCTCTATTCCTGAAATTGATCAG
6. KR021407.1_China ATGTCAGATACTTTTTCAACAACGCTCCCCCAGCTCAAGCTTCAACTATCTATTCCTGAATTAACTCAG
7. KX610809.1_Kenya_maize ATGTCAGAAACTTTTTCAACGATGCTCCCACGTCTGAACATCCAACTCTCTATTCCTGAAATTGATCAG
8. KX830963.1_EastTimor ATGTCAGGTACTTTTTCAACAACGCTCCCCCAGCTCAAGCTTCAACTATCTATTCCTGAATTAACTCAG
9. KX883690.1_China_Spider ATGTCAGGTACTTTTTCAACAACGCTCCCCCAGCTCAAGCTTCAACTATCTATTCCTGAATTAACTCAG
10. KX883929.1_China_Odonata ATGTCAGATACTTTTTCAACAACGCTCCCCCAGCTCAAGCTTCAACTATCTATTCCTGAATTAACTCAG
11. LN907586.1_Kenya_Aphids ATGTCAGAAACTTTTTCAATGATGCTCCCACGTCTGGCGCTTCAACTATCTACTCCTGAAATTAATCAG
12. LN907587.1_Kenya_Aphid ATGTCAGAAACTTTTTCAATGATGCTCCCACGTCTGGCGCTTCAACTATCTACTCCTGAAATTAATCAG
13. LN907588.1_Kenya_Aphid ATGTCAGAAACTTTTTCAATGATGCTCCCACGTCTGGCGCTTCAACTATCTACTCCTGAAATTAATCAG
14. LN907589.1_Kenya_Aphid ATGTCAGAAACTTTTTCAATGATGCTCCCACGTCTGGCGCTTCAACTATCTACTCCTGAAATTAATCAG
15. MF458892.1_Kenya_Maize ATGTCAGGTACTTTTTCAACAACGCTCCCCCAGCTCAAGCTTCAACTATCTATTCCTGAATTAACTCAG
16. MF458893.1_Kenya_Aphid ATGTCAGAAACTTTTTCAATGATGCTCCCACGTCTGGCGCTTCAACTATCTACTCCTGAAATTAATCAG
17. MF795134.1_China_Wasp ATGTCAGGTACTTTTTCAACAATGCTCCCCCAGCTCAAGCTTCAACTATCTATTCCTGAATTAACTCAG
18. MF795135.1_China_Aphid ATGTCAGGTACTTTTTCAACAACGCTCCCCCAGCTCAAGCTTCAACTATCTATTCCTGAATTAACTCAG
19. NC_004365.1_LabIsolates ATGTCAGGTACTTTTTCAACAACGCTCCCCCAGCTCAAGCTTCAACTATCTATTCCTGAGTTAACTCAG
20. SRF35_MK014483_Kenya_Comm... ATGTCAGGTACTTTTTCAACAACGATCCCTCAGCTCAAGCTTCAACTATCTATTCCTGAATTAACTCAG
70 80 90 100 110 120 130
Consensus CTAAAGATACGCGATGCCCAAGCACACGAAAAACCACGACTTGTTGCTCAATTGACTCGGTCTRCTCTA
1. KX884399.1_outgroup TTGAAGATACGTGATGCCCAGACTCAGGAAAAACCACGACTTGTTGCGCAATTGACTCGCTCTGCCCTT
2. AF536531.1_LabIsolates CTAAAGATACGCGATGCCCAAGCACACGAAAAACCAAGAATTGTTGCTCAATTAACTAGATCTACTCTA
3. JQ320375.1_China CTAAAGATACGTGATGCCCAAGCACACGAAAAACCACGACTTGTTGCTCAATTGACTCGGTCTACTCTA
4. JX480861.1_Israel CTGAAGATACGTGATGCACAGGCACATGAACAACCACGAATTGTTGCACAATTGACTCGCTCGGCTCTT
5. KJ817182.1_Kenya_aphids CTAAAGAAACGCGATGTCCAAACAATGGAATCTCCACGATTTGTTCCTCAAATTTCTCAATCTGCTCTT
6. KR021407.1_China CTAAAGATACGCGATGCCCAAGCACACGAAAAACCACGACTTGTTGCTCAATTGACTCGGTCTACTCTA
7. KX610809.1_Kenya_maize CTAAAGAAACGCGATGTCCAAACAATGGAATCTCCACGATTTGTTCCACAAATGACTCAATCAGCTCTT
8. KX830963.1_EastTimor CTAAAGATACGCGATGCCCAAGCACACGAAAAACCTCGACTTGTTGCTCAATTGACTCGGTCTACTCTA
9. KX883690.1_China_Spider CTAAAGATACGCGATGCCCAAGCACACGAAAAACCACGACTTGTTGCTCAATTGACTCGGTCTACCCTA
10. KX883929.1_China_Odonata CTAAAGATACGCGATGCCCAAGCACACGAAAAACCACGACTTGTTGCTCAATTGACTCGGTCTACTCTA
11. LN907586.1_Kenya_Aphids CTGAAGATACGCGATGCACAGGCACACGAACAACCACGAATTGTTGCACAATTGACTCGTTCGGCTCTT
12. LN907587.1_Kenya_Aphid CTGAAGATACGCGATGCACAGGCACACGAACAACCACGAATTGTTGCACAATTGACTCGTTCGGCTCTT
13. LN907588.1_Kenya_Aphid CTGAAGATACGCGATGCACAGGCACACGAACAACCGCGAATTGTTGCACAATTGACTCGTTCGGCTCTT
14. LN907589.1_Kenya_Aphid CTGAAGATACGCGATGCACAGGCACACGAACAACCGCGAATTGTTGCACAATTGACTCGTTCGGCTCTT
15. MF458892.1_Kenya_Maize CTAAAGATACGCGATGCCCAAGCACACGAAAAACCACGACTTGTTGCTCAATTGACTCGGTCTGCTCTA
16. MF458893.1_Kenya_Aphid TTAAAGATACGCGATGCACAGGCACACGAACAACCACGAATTGTTGCACAATTGACTCGTTCGGCTCTT
17. MF795134.1_China_Wasp CTAAAGATACGCGATGCCCAAGCACACGAAAAACCACGACTTGTTGCTCAATTGACTCGGTCTACTCTA
18. MF795135.1_China_Aphid CTAAAGATACGCGATGCCCAAGCACACGAAAAACCACGACTTGTTGCTCAATTGACTCGGTCTACCCTA
19. NC_004365.1_LabIsolates CTAAAGATACGCGATGCCCAAGCACACGAAAAACCAAGAATTGTTGCTCAATTAACTAGATCTACTCTA
20. SRF35_MK014483_Kenya_Comm... CTAAAGATACGCGATGCCCAAGCACACGAAAAACCACGACTTGTTGCTCAATTGACTCGGTCTACTCTA

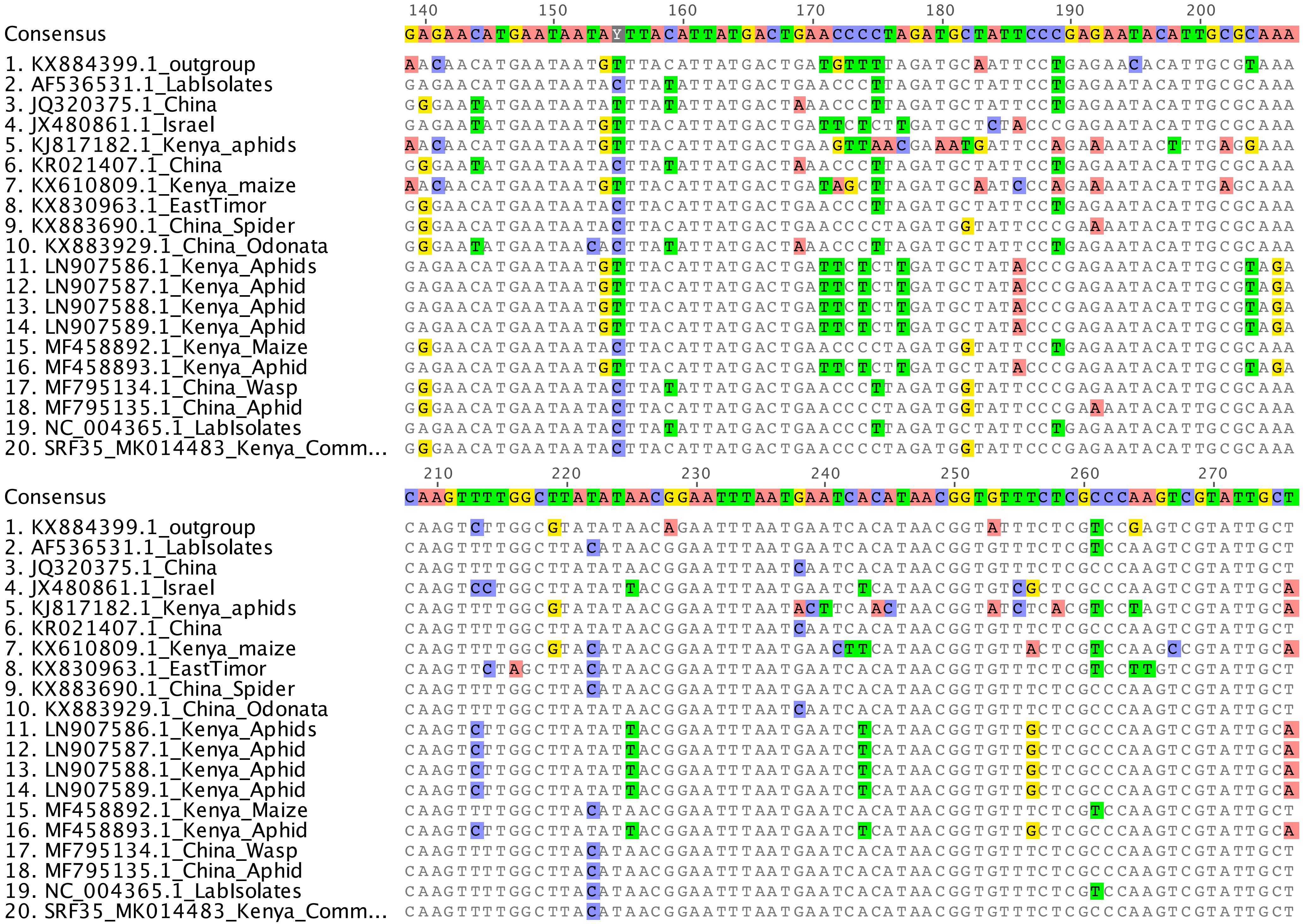

140
150
160
170
180
190
200
Consensus
GAGAACATGAATAATAYTTACATTATGACTGAACCCTAGATGCTATTCCCGAGAATACATTGCGCAAA
1. KX884399.1_outgroup
2. AF536531.1_LabIsolates
3. JQ320375.1_China
4. JX480861.1_Israel
5. KJ817182.1_Kenya_aphids
6. KR021407.1_China
7. KX610809.1_Kenya_maize
8. KX830963.1_EastTimor
9. KX883690.1_China_Spider
10. KX883929.1_China_Odonata
11. LN907586.1_Kenya_Aphids
12. LN907587.1_Kenya_Aphid
13. LN907588.1_Kenya_Aphid
14. LN907589.1_Kenya_Aphid
15. MF458892.1_Kenya_Maize
16. MF458893.1_Kenya_Aphid
17. MF795134.1_China_Wasp
18. MF795135.1_China_Aphid
19. NC_004365.1_LabIsolates
20. SRF35_MK014483_Kenya_Comm...
210
220
230
240
250
260
270
Consensus
CAAGTTTTGGCTTATATAACGGAATTTAATGAATCACATAACGGTGTTTCTCGCCCAAGTCGTATTGCT

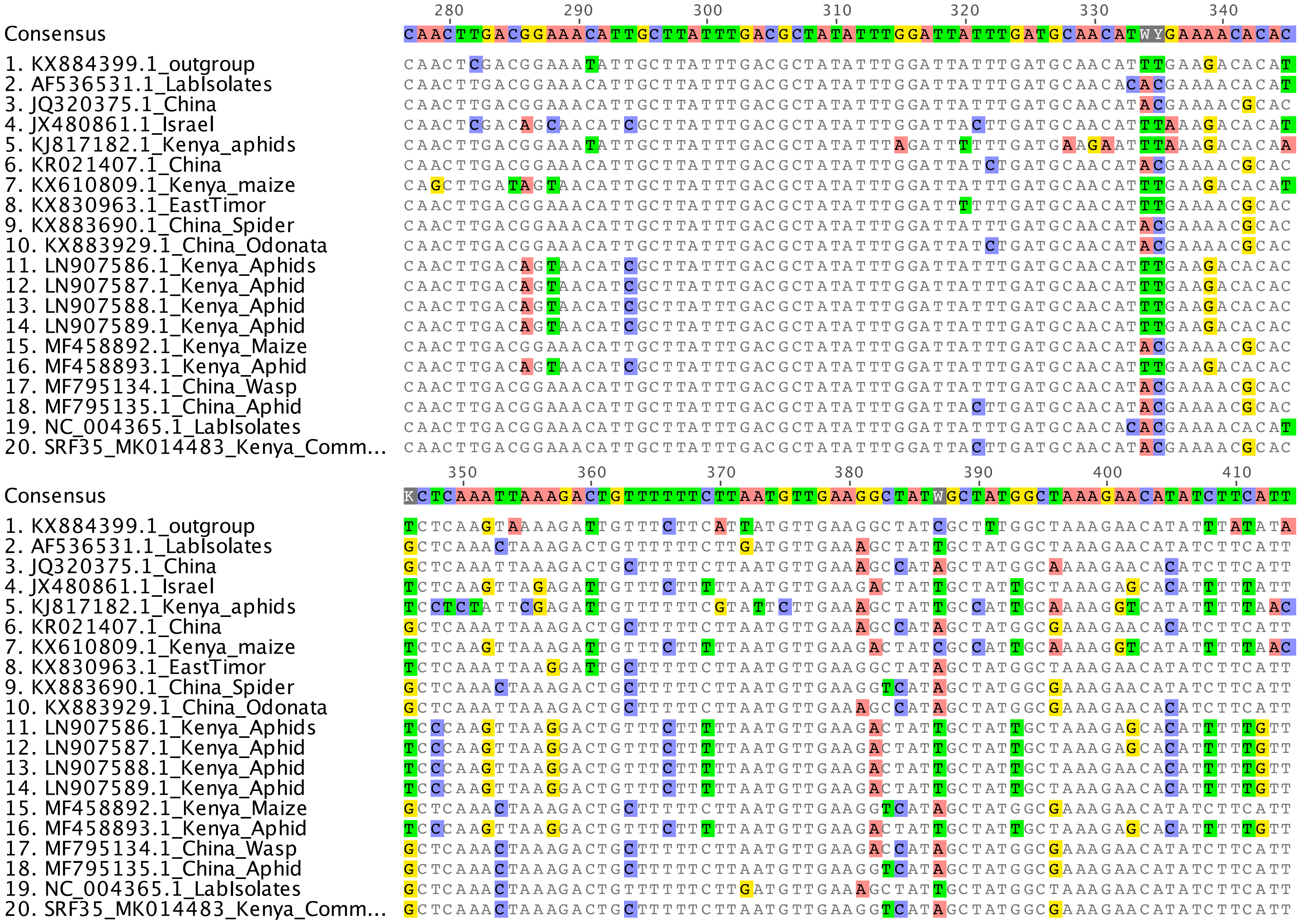
280 290 300 310 320 330 340
Consensus
CAACTTGACGGAAACATTGCTTATTTGACGCTATATTTGGATTATTTGATGCAACATWYGAAAACACAC
1. KX884399.1_outgroup
2. AF536531.1_LabIsolates
3. JQ320375.1_China
4. JX480861.1_Israel
5. KJ817182.1_Kenya_aphids
6. KR021407.1_China
7. KX610809.1_Kenya_maize
8. KX830963.1_EastTimor
9. KX883690.1_China_Spider
10. KX883929.1_China_Odonata
11. LN907586.1_Kenya_Aphids
12. LN907587.1_Kenya_Aphid
13. LN907588.1_Kenya_Aphid
14. LN907589.1_Kenya_Aphid
15. MF458892.1_Kenya_Maize
16. MF458893.1_Kenya_Aphid
17. MF795134.1_China_Wasp
18. MF795135.1_China_Aphid
19. NC_004365.1_LabIsolates
20. SRF35_MK014483_Kenya_Comm...
350 360 370 380 390 400 410
Consensus
KCTCAAATTAAAGACTGTTTTTCTTAATGTTGAAGGCTATWGCTATGGCTAAAGAACATATCTTCATT
1. KX884399.1_outgroup
2. AF536531.1_LabIsolates
3. JQ320375.1_China
4. JX480861.1_Israel
5. KJ817182.1_Kenya_aphids
6. KR021407.1_China
7. KX610809.1_Kenya_maize
8. KX830963.1_EastTimor
9. KX883690.1_China_Spider
10. KX883929.1_China_Odonata
11. LN907586.1_Kenya_Aphids
12. LN907587.1_Kenya_Aphid
13. LN907588.1_Kenya_Aphid
14. LN907589.1_Kenya_Aphid
15. MF458892.1_Kenya_Maize
16. MF458893.1_Kenya_Aphid
17. MF795134.1_China_Wasp
18. MF795135.1_China_Aphid
19. NC_004365.1_LabIsolates
20. SRF35_MK014483_Kenya_Comm...

```
                                  420       430       440       450       460       470       480
Consensus                     GAAGATGGGTCCTTTTACTCTATAGCTATWGCTCTTCGTTTAATAAACGCATCATTGGCAACGATTTTA

1. KX884399.1_outgroup        AAACATGAAATGTTCCACTCTATGGCAATATCCCTTCGTTTAATAAACGCATCATTGGCTATGATTTTA
2. AF536531.1_LabIsolates      GAAGATGGGTCCTTTTACTCCATAGCTATTGCTCTTCGCTTAATAAACGCATCATTGGCAACGATTTTA
3. JQ320375.1_China            GAGGATGGGTCCTTTTATTCTATAGCTATCGCTCTTCGTTTAATAAACGCATCATTGGCAACGATTTTG
4. JX480861.1_Israel           AAAAATGACACTTTTTATTCAATGGCTATATCTCTTCGTTTAATAAACGCATCATTGGCAACGATTTTA
5. KJ817182.1_Kenya_aphids     AAAGATGATCAGTTTTTCTCAATGGCGATATCTTTACGTTTAATAAACGCATCATTGGCTACGATTTTA
6. KR021407.1_China            GAGGATGGGTCCTTTTATTCTATAGCTATTGCTCTTCGTTTAATAAACGCATCATTGGCAACGATTTTG
7. KX610809.1_Kenya_maize      AAAGATGATCACTTTTACTCAATAGCGATAGCTCTACGTTTAATAAACGCATCATTGGCAACGATTTTA
8. KX830963.1_EastTimor        GAAGACGGTTCTTTTTACTCCATAGCTATCGCTCTTCGTTTAATAAACGCATCCTTGGCAACGATTTTG
9. KX883690.1_China_Spider     GAAGATGGATCCTTTTACTCTATAGCTATTGCTCTTCGTTTAATAAACGCATCATTGGCAACGATTTTG
10. KX883929.1_China_Odonata   GAGGATGGGTCCTTTTATTCTATAGCTATTGCTCTTCGTTTAATAAACGCATCATTGGCAACGATTTTG
11. LN907586.1_Kenya_Aphids    AAAGATGACACTTTTTACTCAATGGCTATATCTCTTCGTTTAATAAACGCATCATTGGCAACGATTTTA
12. LN907587.1_Kenya_Aphid     AAAGATGACACTTTTTACTCAATGGCTATATCTCTTCGTTTAATAAACGCATCATTGGCAACGATTTTA
13. LN907588.1_Kenya_Aphid     AAAGATGACACTTTTTACTCAATGGCTATATCTCTTCGTTTAATAAACGCATCATTGGCAACGATTTTA
14. LN907589.1_Kenya_Aphid     AAAGATGACACTTTTTACTCAATGGCTATATCTCTTCGTTTAATAAACGCATCATTGGCAACGATTTTA
15. MF458892.1_Kenya_Maize     GAAGATGGGTCCTTTTACTCTATAGCTATTGCTCTTCGTCTAATAAACGCATCATTGGCAACGATTTTG
16. MF458893.1_Kenya_Aphid     AAAGATGATACTTTTTACTCAATGGCTATATCTCTTCGTTTAATAAACGCATCATTGGCAACGATTTTA
17. MF795134.1_China_Wasp      GAAGATGGGTCCTTTTACTCTATAGCTATTGCTCTTCGTTTACTAAACGCATCATTGGCAACGATTTTG
18. MF795135.1_China_Aphid     GAAGATGGGTCCTTTTACTCTATAGCTATTGCTCTTCGTTTAATAAACGCATCATTGGCAACGATTTTG
19. NC_004365.1_LabIsolates    GAAGATGGGTCCTTTTACTCCATAGCTATTGCTCTTCGCTTAATAAACGCATCATTGGCAACGATTTTA
20. SRF35_MK014483_Kenya_Comm... GAAGATGGGTCCTTTTACTCTATAGCTATTGCTCTTCGTTTAATAAACGCATCACTGGCAACGATTTTG
```

```
                                   490 493
Consensus                     AACAAACCCG

1. KX884399.1_outgroup        AACACACCCG
2. AF536531.1_LabIsolates      AACAAACCCG
3. JQ320375.1_China            AACAAACCCG
4. JX480861.1_Israel           AACAAACCTG
5. KJ817182.1_Kenya_aphids     AACAACCCCG
6. KR021407.1_China            AACAAACCCG
7. KX610809.1_Kenya_maize      AACAAACCCG
8. KX830963.1_EastTimor        AACAAACCCG
9. KX883690.1_China_Spider     AACAAACCCG
10. KX883929.1_China_Odonata   AACAAACCCG
11. LN907586.1_Kenya_Aphids    AATAAACCCG
12. LN907587.1_Kenya_Aphid     AATAAACCCG
13. LN907588.1_Kenya_Aphid     AATAAACCCG
14. LN907589.1_Kenya_Aphid     AATAAACCCG
15. MF458892.1_Kenya_Maize     AACAAACCCG
16. MF458893.1_Kenya_Aphid     AACAAACCCG
17. MF795134.1_China_Wasp      AACAAACCCG
18. MF795135.1_China_Aphid     AACAAACCCG
19. NC_004365.1_LabIsolates    AACAAACCCG
20. SRF35_MK014483_Kenya_Comm... AACAAACCCG
```
